# Understanding developmental and adaptive cues in pine through metabolite profiling and co-expression network analysis

**DOI:** 10.1093/jxb/erv118

**Published:** 2015-04-01

**Authors:** Rafael A. Cañas, Javier Canales, Carmen Muñoz-Hernández, Jose M. Granados, Concepción Ávila, María L. García-Martín, Francisco M. Cánovas

**Affiliations:** ^1^Departamento de Biología Molecular y Bioquímica, Facultad de Ciencias, Universidad de Málaga, Campus Universitario de Teatinos s/n, 29071 Málaga, Spain; ^2^Unidad de Nanoimagen, Centro Andaluz de Nanomedicina y Biotecnología (BIONAND), Parque Tecnológico de Andalucía, C/ Severo Ochoa 35, 29590 Campanillas (Málaga), Spain

**Keywords:** Adaptation, development, gene co-expression analysis, metabolite profiling, needles, *Pinus pinaster*.

## Abstract

Conifers include long-lived evergreen trees of great economic and ecological importance, including pines and spruces. During their long lives conifers must respond to seasonal environmental changes, adapt to unpredictable environmental stresses, and co-ordinate their adaptive adjustments with internal developmental programmes. To gain insights into these responses, we examined metabolite and transcriptomic profiles of needles from naturally growing 25-year-old maritime pine (*Pinus pinaster* L. Aiton) trees over a year. The effect of environmental parameters such as temperature and rain on needle development were studied. Our results show that seasonal changes in the metabolite profiles were mainly affected by the needles’ age and acclimation for winter, but changes in transcript profiles were mainly dependent on climatic factors. The relative abundance of most transcripts correlated well with temperature, particularly for genes involved in photosynthesis or winter acclimation. Gene network analysis revealed relationships between 14 co-expressed gene modules and development and adaptation to environmental stimuli. Novel Myb transcription factors were identified as candidate regulators during needle development. Our systems-based analysis provides integrated data of the seasonal regulation of maritime pine growth, opening new perspectives for understanding the complex regulatory mechanisms underlying conifers’ adaptive responses. Taken together, our results suggest that the environment regulates the transcriptome for fine tuning of the metabolome during development.

## Highlight

This work highlights the complex interaction between developmental processes and environmental adaptations of maritime pine trees growing under natural conditions.

## Introduction

Trees account for nearly 80% of total plant biomass (Olson *et al.*, 1983) and 50–60% of annual net primary production in terrestrial ecosystems (Field *et al.*, 1998). Coniferous forests make important contributions since they dominate major regional ecosystems, particularly in the northern hemisphere (Farjon, 2010). They also have great economic importance as primary sources of timber and paper production globally. Conifers have evolved distinct developmental, metabolic, and physiological adaptations since their divergence from a putative common ancestor shared with angiosperms over 300 million years ago (Bowe *et al.*, 2000). Most species are long-living evergreens (Farjon, 2010) with extremely large genomes ranging from 20 to 40 Gb (Mackay et al., 2012; Ritland, 2012).

Key variables that govern perennial woody plant growth include both diurnal and seasonal changes in environmental conditions. Evergreen conifer needles typically live for several years, so they need to survive the winter period when they are subjected to cold, drought, and oxidative stresses, and photosynthetic rates must be reduced due to the low temperatures (Öquist and Huner, 2003). Essential responses to these environmental changes include accumulation of ‘compatible solutes’ to maintain protein stability and cellular integrity under frost stress (Galindo-González *et al.*, 2012; Simard *et al.*, 2013), with adjustments of membrane lipid composition to maintain their fluidity (Roden *et al.*, 2009). Clearly, when photosynthesis is reduced cell energy requirements must be met through metabolic pathways such as glycolysis or oxidative phosphorylation (Ophir *et al.*, 2009; Grene *et al.*, 2012; Galindo-González *et al.*, 2012; Collakova *et al.*, 2013). Accordingly, photosynthetic rates and capacities of adult needles are temperature- and/or photoperiod-dependent, peaking in the summer (Öquist and Huner, 2003).

Like all plants, conifers also respond in complex fashions to numerous other biotic and abiotic factors that may vary diurnally, seasonally, or unpredictably in their environments. To understand these responses fully they must be considered in conjunction with inherent developmental changes. Notably, needles’ photosynthetic capacity increases as they mature, peaks when they are fully expanded, then gradually declines until senescence, typically several years later (Warren, 2005). Senescence is a tightly controlled process in which various nutrients may be mobilized; for example, Rubisco (EC: 4.1.139) and other soluble proteins in older leaves may be used as nitrogen stores during the development of new needles in the spring (Gezelius and Hallén, 1980; Näsholm and Ericsson 1990). Distinguishing between developmental changes mediated by internal processes and responses to environmental fluctuations in conifers is clearly important, but challenging, since they involve many interactions. Fortunately, advances in next-generation sequencing and the development of powerful software have enormously accelerated genomic analysis of conifers. Recently reference transcriptomes have been established for *Picea sitchensis* (Ralph *et al.*, 2008), *Picea glauca* (Rigault *et al.*, 2011), and *Pinus pinaster* (Canales *et al.*, 2014). Assemblies of genome drafts of *Picea abies* (Nystedt *et al.*, 2013), *Picea glauca* (Birol *et al.*, 2013), and *Pinus taeda* (Zimin *et al.*, 2014) have also recently been reported.

Maritime pine (*Pinus pinaster* L. Aiton) is a conifer species with great economic and environmental value that is widely distributed in the south-western area of the Mediterranean region, dominating forests in France, Portugal, and Spain. It has high phenotypic plasticity, accompanied by high tolerance to abiotic stresses such as drought, and hence it is distributed in widely varying environments at altitudes ranging from sea level to 2000 m (Aranda *et al.*, 2010; Gaspar *et al.*, 2013). Abundant genomic resources and phenotypic data for the species, in addition to its reference transcriptome, are also now available (Canales *et al.*, 2014; http://www.scbi.uma.es/sustainpine, last accessed 11 March 2015; https://w3.pierroton.inra.fr/PinusPortal, last accessed 11 March 2015).

The present study is a systems-based approach to develop further studies on the relationship between maritime pine and the environment. In this work, we examined metabolite and transcriptomic profiles of needles from the four most recent whorls of maritime pine trees growing in a forest at 1245 m altitude, under natural conditions, during the course of a year. The results indicate that major components of their metabolite profiles were strongly affected by inherent acclimation to winter (notably sucrose and pinitol) and needle age (e.g. betaine and methionine). In contrast, gene expression profiles were mainly dependent on environmental variables (notably the expression of genes involved in photosynthesis and winter acclimation being positively and negatively correlated with temperature, respectively). Gene co-expression analysis identified 14 eigengene modules mainly correlated with temperature except for one module which is correlated with needle development and contains genes encoding enzymes of the phenylpropanoid, flavonoid, and terpenoid synthesis pathways. We also identified two genes that may participate in the regulation of these pathways during needle development and/or responses to environmental cues. The acquired knowledge should enhance understanding of gene function and adaptation in maritime pine and other economically and environmentally important gymnosperms (Neale and Kremer, 2011).

## Materials and methods

### Plant material

**Fig. 1.**
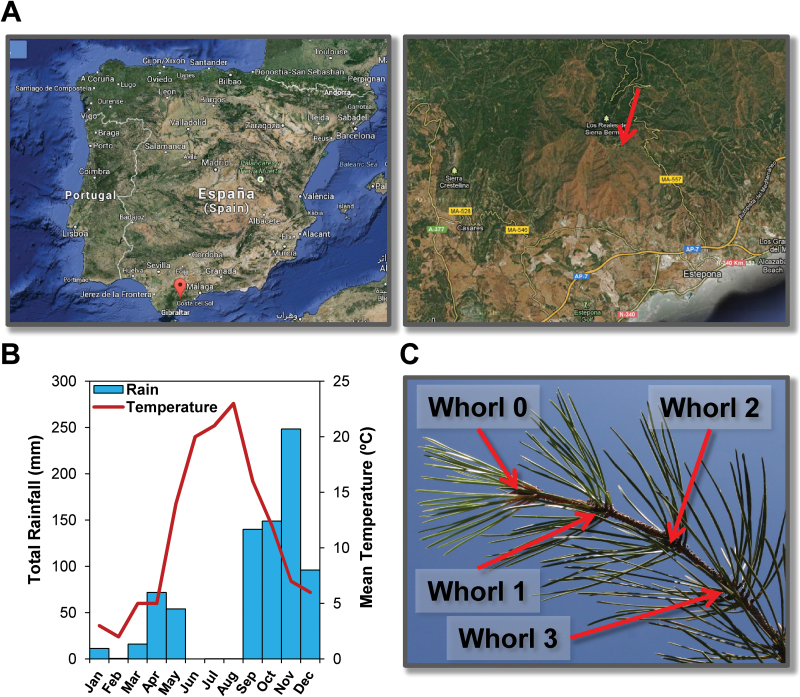
Location and variables of sampling. (A) Sampling location. (B) Temperature and rainfall at the sampling location during 2012. (C) Maritime pine branch with the four whorls isolated in the present work. This figure is available in colour at *JXB* online.

*Pinus pinaster* needles from 25-year-old trees were sampled at Los Reales de Sierra Bermeja (Estepona, Spain) (30S X:303.095 Y:4.039.618) at 1245 m altitude on the south side of the mountain (Fig. 1A). The harvesting of needle whorls was carried out every month during 2012, from January to December, in six independent trees. Harvesting dates are given in Supplementary Table S1. A total of 264 samples were obtained. Three whorls were considered from January to April, and four whorls for the rest of the months due to the emergence of a new whorl in May (Fig. 1C). As in *P. pinaster* each needle whorl corresponds to the annual growth of a single year; the whorls were named by numbers: ‘0’ for the new needles of 2012, ‘1’ for 2011, ‘2’ for 2010, and ‘3’ for 2009. The harvested needles were always those at 3–4 m height in the trees, on the south side. The samplings were carried out 4–5 hours after sunrise. Isolated needles were immediately frozen in liquid N and subsequently placed in dry ice for transport to the laboratory. All frozen samples were reduced to a homogenous powder with a Mixer Mills MM400 (Retsh, Haan, Germany) and stored at –80ºC until further use for metabolite and RNA extraction. The meteorological data (monthly average temperature and rainfall) were provided by the ‘Sistema Automático de Información Hidrológica (SAIH) para Los Reales’ (30S X: 302.233 Y: 4.040.118) (Fig. 1B).

### Metabolite extraction and NMR measurements

The metabolites for ^1^H-NMR analysis were extracted following the protocol described by Kruger *et al.* (2008). For each sample, 0.1g of frozen powder was used for extraction.

The ^1^H-NMR analyses were carried out on a Bruker ASCEND™ 400 MHz NMR Spectrometer. 1D ^1^H-NMR spectra were acquired using the composite pulse sequence *zgcppr* with 1 s water pre-saturation delay, 8kHz spectral width, 32 k data points, 16 scans, and a recycle time of 8 s. Quantitative analysis of the NMR spectra was performed using the software LCModel (Linear Combination of Model Spectra) (Provencher, 1993) with our own reference metabolite spectra library (Supplementary Data S1) and an electronically generated signal, ERETIC (Electronic Reference To Access *In vivo* Concentrations), as an internal reference (Akoka *et al.*, 1999). Metabolites could be determined at millimolar concentrations in the final solution. The analysis of the metabolite data was carried out using the R package WGCNA as proposed by DiLeo *et al.* (2011). The script employed is in Supplementary Method S1. The principal component analysis (PCA) was made using the R function *prcomp*. The missing data were replaced with the median. The PCA script employed is in Supplementary Method S2.

### RNA extraction

Total RNA was isolated following the protocol described by Liao *et al.* (2004) and modified by Canales *et al.* (2012). The RNA concentration and purity were determined spectrophotometrically using A260/A280nm and A260/A230nm ratios. RNA quality was further confirmed by agarose gel electrophoresis.

### RNA amplification, sample labelling, and microarray hybridization

A cDNA microarray was used (PINARRAY2) for the gene expression measures of the total RNA samples. This is the second version of the original microarray presented in Villalobos *et al.* (2012). In the PINARRAY2 8208 spots were included as well as negative and positive controls corresponding to cDNAs in SustainPineDB (Canales *et al.*, 2014). The spots were duplicated in two fields per slide. A reference transcriptome of *P. pinaster* was not available at the time that this work was initiated, therefore PINARRAY2 was used as a reliable tool instead RNA-seq.

For the microarray hybridization, the samples from January, March, May, July, September, and November were used. Before the RNA amplification and labelling, the RNA samples were pooled two by two to obtain three working replicates for microarray hybridization as presented in Supplementary Figure S1. A total of 66 microarrays were hybridized. As much as 500ng total RNA extracted from needle samples was amplified using the TargetAmp™ 1-Round aRNA Amplification Kit (Epicentre, WI, USA) following the manufacturer’s instructions. The antisense amplified RNA (aRNA) was labelled with the CyDye Post-Labelling Reactive Dye Pack (GE Healthcare, Barcelona, Spain). In the microarray experimental design we used a pool of January RNA samples as a common reference sample for data normalization. The reference sample was labelled with Cy5 and the target samples with Cy3. For the CyDye labelling, 5 μl of coupling buffer (0.2M sodium bicarbonate) and 10 μl of CyDye were added to 10 μg of aRNA in a 5 μl volume. Then, the mix was incubated in darkness for 90min at room temperature. Finally 7.5 μl of 4M hydroxylamine was added and the solution was incubated in darkness for a further 15min at room temperature. The CyDye-labelled aRNA was purified with the NucleoSpin® Gel and PCR Clean-up kit (Macherey Nagel, Düren, Germany) with a single elution of 20 μl. The quantity and quality of the labelling was determined using a Nanodrop spectrophotometer ND-1000 (Nanodrop, DE, USA).

Before the microarray hybridizations, the slides were pre-incubated for 1h at 45ºC in a solution with 5X SSC buffer, 1% BSA, and 0.1% SDS. Then, the slides were washed twice with 0.01 SSC buffer for 30 s and dried in a centrifuge for 2min at 2000*g*. Before the hybridization, 40 μl of hybridization solution [62.5% (v/v) formamide, 6.25X SSC, 0.125mg ml^–l^ salmon sperm, 0.625% BSA, 0.125% SDS, 12.5 μg ml^–l^ polyA] was added to a 10 μl solution containing 2 μg of the problem sample labelled with Cy3 and 2 μg of the reference sample labelled with Cy5. The mix was denatured at 65ºC for 10min in a thermal cycler. After this the sample mix was loaded into a PINARRAY2 slide and covered with a HybriSlip^TM^ hybridization cover (Grace Bio-Labs, OR, USA). Microarray hybridization was performed in a Genetix Hybridization Chamber (Genetix, New Milton, UK) at 42ºC for 16h. Hybridized slides were scanned at 5 μm resolution and their signal intensities were detected by a GenePix 4100A microarray scanner (Molecular devices, Sunnyvale, CA, USA). The raw data were subsequently preprocessed and normalized with the R package for the common reference design microarray BABAR (Alston *et al.*, 2010) (Supplementary Method S3). The data discussed in this publication have been deposited in NCBI’s Gene Expression Omnibus (Edgar *et al.*, 2002) and are accessible through GEO Series accession number GSE55868 (http://www.ncbi.nlm.nih.gov/geo/query/acc.cgi?acc=GSE55868, last accessed 12 March 2015).

### Gene co-expression network analysis

The gene co-expression network analyses were carried out using the R package WGCNA (Langfelder and Horvath, 2008). The WGCNA scripts used are in Supplementary Method S4. Genes with too many missing values were eliminated (343 genes) (Supplementary Data S2) and the sample *w2rep2november* was also removed after outlier verification through hierarchical clustering. Before network construction the proper soft-thresholding power was determined by performing an analysis of the network topology. We use the one-step network construction option with a soft-thresholding power value of 14. Genes with a kME >0.3 were assigned to an eigengene module or to a grey module if they did not meet the above criteria. The connectivity and module membership for each gene are in Supplementary Data S3. The gene network screening was made with weighted and Pearson correlations using the WGCNA *networkScreening* function. The *q*-values (FDR) were calculated (Storey *et al.*, 2004) and correlations were considered significant with a *q*-value <0.05. Correlations of the mean gene expression of the modules and the individual expression of the genes were performed against the environmental variables; former monthly mean temperature (Temperature) and accumulated precipitation (Rain), respectively. For the ageing and/or developmental variable the whorl provenance of the samples (Whorl) was used for correlations. After initial approximations, gene expression was mainly adjusted to long-term dynamics of temperature (Supplementary Figure S2).

### Functional gene-enrichment analyses

The orthologous assignment and pathway mapping of the genes from the modules were made with the KEGG Automatic Annotation Server (KAAS, Moriya *et al.*, 2007) using the SBH option. Gene ontology (GO) terms assignment was based on homology to the *Arabidopsis* TAIR protein database using BLASTX (Altschul *et al.*, 1997). The Singular Enrichment Analysis (SEA) of the GO terms was made in the AGRIGO web tool with standards parameters (Du *et al.*, 2010). The mapping file for the Mapman representation was made using the Mercator web server (Lohse *et al.*, 2014). The Mapman image was made using the Mapman software (Thimm *et al.*, 2004).

### RT-qPCR analyses

Validation of the gene expression of the microarray results was carried out by RT-qPCR. The RT-qPCRs were as described previously by Cañas et al. (2014). The primers used for the RT-qPCR reactions are presented in Supplementary Table S2. The raw fluorescence data from each reaction was fitted to the MAK2 model, which requires no assumptions about the amplification efficiency of the RT-qPCR assay (Boggy and Woolf, 2010). The initial target concentrations (D0 parameter) for each gene were deduced from the MAK2 model using the qpcR package for the R environment (Ritz and Spiess, 2008) and normalized to the geometric mean of two reference genes. Actin and a 40S ribosomal protein S27 were used as references for validation. For the RT-qPCR analysis, three biological replicates and three technical replicates were made per sample.

## Results

### Climate conditions

Because of the altitude of the sampling area, tree phenology was delayed by ~1 month in comparison to trees near sea level. Male cones appeared in April and the new needle expansion began in May. The new needles were fully expanded between August and September. The formation of male cones and new needle expansion were coincident with the increase in temperature and spring rainfall. There is no complete climate series (30 years records) for temperature and rainfall at Los Reales (only since 2007 for temperature and since 1994 for rainfall). However, temperature and rainfall records for 2012 were similar to those usually registered in the sampling area with no significant deviations except for the winter months (January to March) with lower precipitation levels (Supplementary Figure S3). Thus, climate data can be considered within the normal range.

### Metabolite network analysis

Analysis of the ^1^H-NMR spectra in samples from needles of maritime pine trees allowed us to quantify 39 different metabolites, including a non-identified sugar (unknown_sugar) (Supplementary Data S1). The spectra of certain metabolites were difficult to separate, notably myo-inositol from glycine (Ins-Gly) and glycerophosphocholine from choline (GPC_Cho). Therefore, both individual and combined quantifications of these metabolites were used in the analyses (Supplementary Data S1). The metabolite profiles were subjected to weighted co-expression network analysis, using the WGCNA package (Langfelder and Horvath, 2008), which groups metabolites into ‘eigengene modules’ (hereafter simply ‘modules’) with highly correlated expression patterns (DiLeo *et al.*, 2011). As shown in the resulting network (Supplementary Figure S4A), most metabolites identified formed part of a single module (colour-coded turquoise). Exceptions included myo-inositol and Ins-Gly (assigned to the brown module); glycerol and hydroxyproline (blue module); and D-mannose, shikimate, L-arginine, D-xylose, L-aspartate, glucose-6-phosphate, and L-proline (grey module, reserved for unassigned metabolites). Supplementary Figure S4B shows Pearson correlations among metabolite modules and the focal variables (Whorl, Temperature, and Rain). Whorl was the most influential variable for most metabolites; all the modules showed significant correlations with it, and all except the blue module very significant correlations. However, only the turquoise module was very significantly correlated with Temperature. Correlations between individual metabolites and the variables tested are shown in Table 1 and Supplementary Data S4. The number of metabolites with significant correlations with Whorl was 31, of which 23 were correlated with Temperature and only five were correlated with Rain. Moreover, the *q*-values of correlations with Whorl were much lower, and the correlation coefficients much higher, than the others. A group of metabolites including betaine, L-leucine, L-methionine, choline, glycerophosphocholine, and sucrose were most strongly correlated with both Whorl and Temperature, but with opposite senses (Table 1).

**Table 1.** Metabolites most correlated with Whorl and Temperature

Metabolites most correlated with Whorl			
Metabolite	WGCNA module	q-value	Correlation
L-Leucine	Turquoise	<4.44E–16	–0.77
L-Methionine	Turquoise	<4.44E–16	–0.71
Choline	Turquoise	<4.44E–16	–0.70
Betaine	Turquoise	<4.44E–16	–0.70
Glycerophosphocholine	Turquoise	<4.44E–16	–0.67
GPC_Cho	Turquoise	<4.44E–16	–0.63
Glutathione	Turquoise	<4.44E–16	–0.62
Sucrose	Turquoise	<4.44E–16	0.51
D-Glucose	Turquoise	<4.44E–16	0.71
D-Glucose 1-phosphate	Turquoise	<4.44E–16	0.75
Metabolites most correlated with Temperature			
Metabolite	WGCNA module	q-value	Correlation
Sucrose	Turquoise	<1.17E–12	–0.67
Creatine	Turquoise	1.17E–12	0.55
Betaine	Turquoise	8.56E–12	0.43
Glycerophosphocholine	Turquoise	3.07E–11	0.42
Choline	Turquoise	1.86E–10	0.40
L-Methionine	Turquoise	1.86E–10	0.44
GPC_Cho	Turquoise	2.65E–10	0.39
L-Isoleucine	Turquoise	5.11E–10	0.42
L-Threonine	Turquoise	2.69E–09	0.41
Succinate	Turquoise	3.30E–09	0.59

**Fig. 2.**
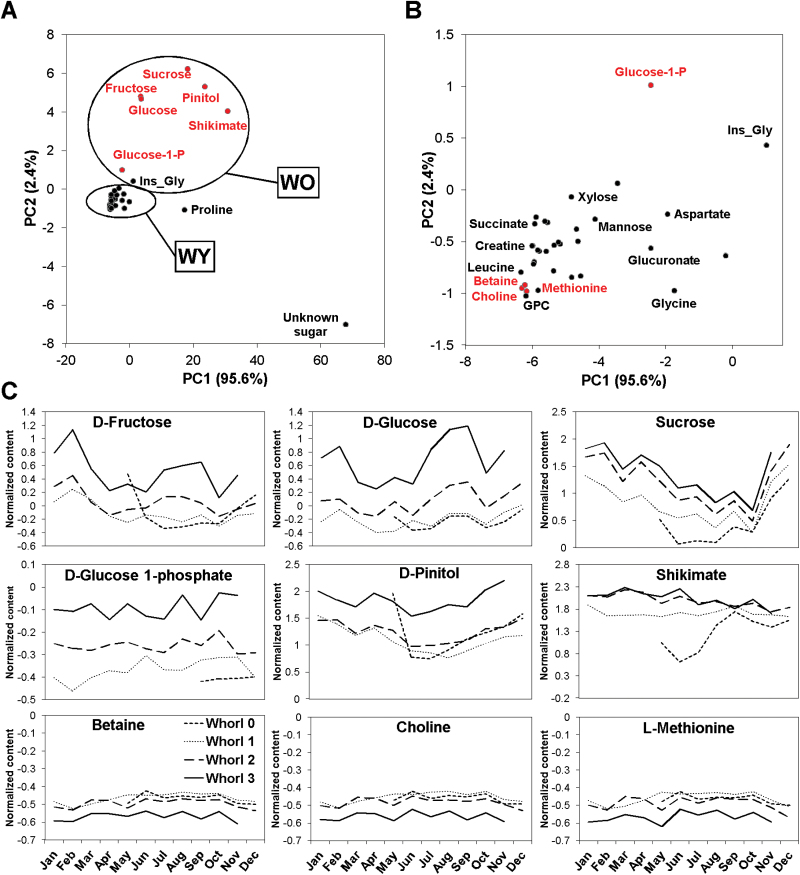
PCA of metabolite profiles. (A) PCA score plot of the identified metabolites. Principal components 1 and 2 explain 95.6 and 2.4% of the total detected variance, respectively, and reveal groups of metabolites that accumulate in older (WO) and younger (WY) whorls. (B) Detail of the WY metabolite area of the PCA plot. (C) Profiles of seven metabolites that accumulated in older whorls (D-fructose, D-glucose, sucrose, D-pinitol, shikimate, D-glucose-1-phosphate, and unknown_sugar), and three that accumulated in younger whorls (betaine, choline, and L-methionine), showing strong significant correlations with Whorl. Whorls 0, 1, 2, and 3 are represented by dashed, dotted, broken, and continuous lines, respectively. The data are means of six values from the NMR spectra. This figure is available in colour at *JXB* online.

Results of PCA of the metabolite profiles are shown in Fig. 2A and B. Most metabolites are concentrated in the same area of the PCA (WY) and only seven of them are displaced from this position (D-fructose, D-glucose, sucrose, D-pinitol, D-glucose-1-phosphate, shikimate, and unknown_sugar). Relative abundance profiles of these seven metabolites are also shown in Fig. 2C. The higher levels of all of them, except unknown_sugar, were in the older whorls (Fig. 2A and C), and thus positively correlated with Whorl as defined here (Table 1, Supplementary Data S4). Fig. 2C also shows profiles of three metabolites (betaine, choline, and L-methionine) placed in the centre of the PCA score plot, showing strong negative correlations with Whorl (Table 1, Supplementary Data S4). They presented similar profiles but lower levels in whorl 3 than in the other whorls. Generally, the metabolite profiles were similar for all the whorls except Whorl 0 (Fig. 2C).

### Gene co-expression network analysis

**Fig. 3.**
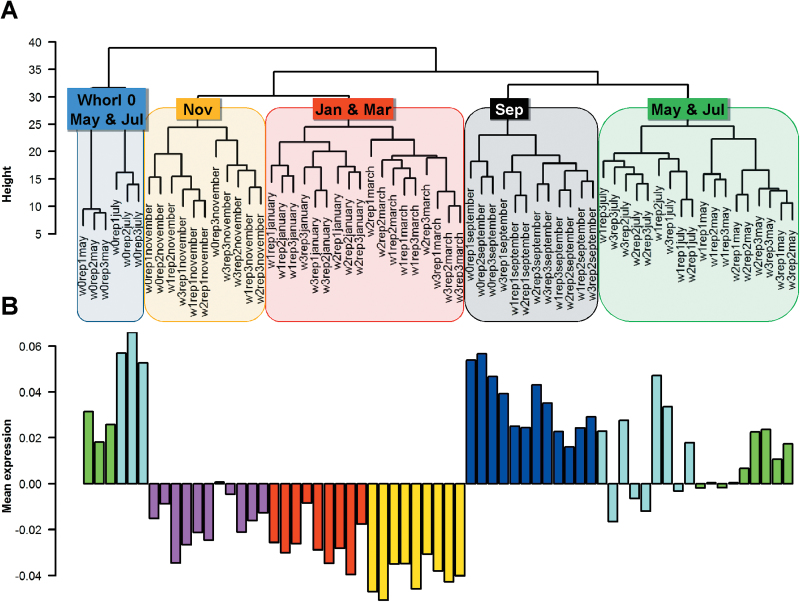
Hierarchical clustering and mean transcript levels in the needle RNA microarray samples. (A) Hierarchical clustering of the microarray samples. The coloured boxes show the harvesting time of each needle sample. The names of the samples (e.g. w0rep1may) indicate the whorl number (w0, Whorl 0), the biological replicate (rep1, individual 1), and the harvesting month. (B) Mean transcript abundance in each sample. Colour code for sampling month: red, January; yellow, March; green, May; light blue, July; dark blue, September; purple, November.

Gene expression profiling was also performed using the same samples as in the metabolite profiling. Hierarchical clustering mainly grouped the samples by season (Fig. 3A): samples collected in January, March, and November clustered more closely than samples collected in May, July, and September (which largely formed a distinct group, except for samples of Whorl 0 from May and July, which formed a separate cluster). Fig. 3B shows that the mean gene expression of the samples was much higher in the samples from May, July, and September than in the other samples. Negative values of normalized gene expression were observed for samples from November, January, and March (Fig. 3B).

**Fig. 4.**
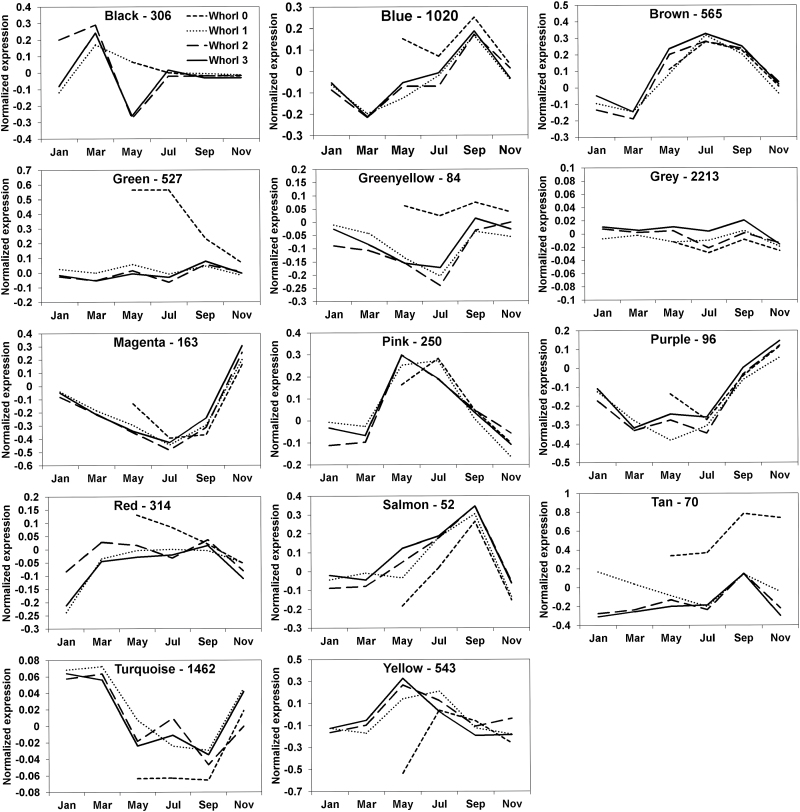
Mean expression profile of genes in each module. The gene expression profile for each whorl during the year is shown for each eigengene module. The number of genes included in each module is shown by its name. Whorls 0, 1, 2, and 3 are represented by dashed, dotted, broken, and continuous lines. Data are means of three values from the normalized microarray data.

The WCGNA analysis identified 14 gene modules each of which was designated with a colour: black, blue, brown, green, green-yellow, grey, magenta, pink, purple, red, salmon, tan, turquoise, and yellow (Supplementary Figure S5). The grey module was reserved for unassigned genes and does not represent a real module. The genes assigned to each module are listed in Supplementary Data S5 and the number of genes in each module and their mean expression levels are presented in Fig. 4 and Supplementary Figure S6. The turquoise and salmon modules were the largest and smallest, containing 1462 and just 52 genes, respectively. In total, 2213 genes were grouped in the grey module. Expression of the genes in the modules was mainly influenced by Temperature. Whorls 1 to 3 displayed similar transcript profiles, which differed substantially from the profiles of juvenile Whorl 0, especially in May and July. In the green and tan modules the differences observed in gene expression in respect to Whorl 0 were more extended in time. Two main temperature-related gene expression profiles were detected: one with higher levels from May to September than in November to March (blue, brown, pink, red, salmon, and yellow modules), and the other with the opposite profile (green-yellow, magenta, purple, and turquoise modules).

### Relationship between gene co-expression modules and traits

**Fig. 5.**
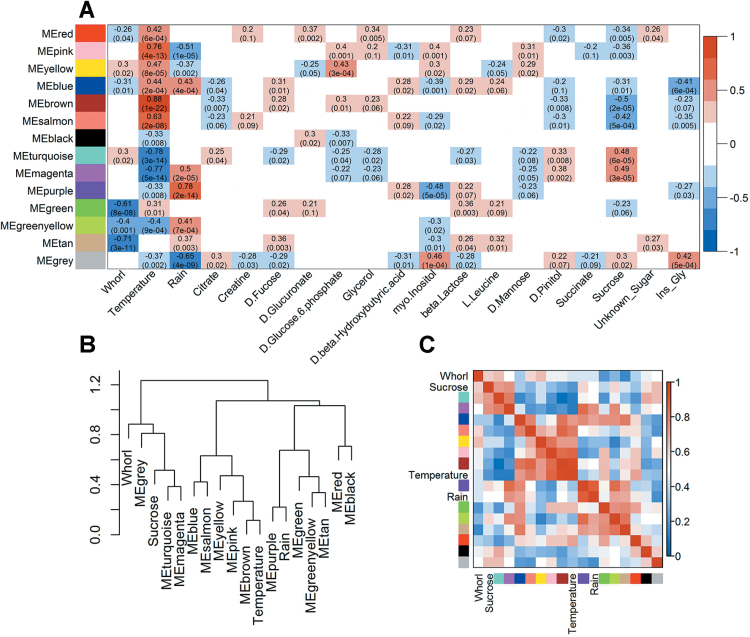
Correlations between eigengene modules, traits, and inter-module relationships. (A) Pearson correlations between the eigengene modules and metabolites, and Whorl, Temperature, and Rain, with *P*-values in parentheses. Only traits with at least one significant correlation (*P* < 0.05) with a module are presented. (B) Eigengene dendrogram. (C) Adjacency heatmap. Colours in the name positions refer to the modules’ names. Colours in the matrix boxes show the magnitude and direction of the correlations: intense blue and red indicate strong negative and positive correlations, respectively. High temperature is positively correlated with high gene expression. Older whorls are positively correlated with high levels of gene expression.

Pearson correlations between the modules and specific traits are summarized in Fig. 5A. The traits are related to measurable variables intrinsic to each sample as the needle age, indicated by whorl, or the rainfall level in the month of the sample harvesting. Nineteen variables (Whorl, Temperature, Rain, and 16 metabolites) were significantly correlated (*P <* 0.05) with at least one module. The traits with higher significant correlations were Whorl, Temperature, Rain, and Sucrose. Whorl was highly correlated with the green, green-yellow, and tan modules (*P*-value < 0.01), but most strongly correlated with the tan module (0.71, *P*-value 3e-11). Temperature also had highly significant (*P*-value < 0.01) correlations with all the modules except the tan module, and was most strongly correlated with the brown module (0.88, *P*-value 1e-22). Rain had very significant correlations (*P*-value < 0.01) with the blue, green-yellow, magenta, pink, purple, tan, yellow, and grey modules, and the strongest correlation with the purple module (0.78, *P*-value 2e-14). Finally, sucrose content had highly significant correlations (*P*-value < 0.01) with the blue, brown, magenta, pink, red, salmon, and turquoise modules, and was most strongly correlated with the brown module (–0.5, *P*-value 2e-05). The relationships between the modules were also examined by generating a dendrogram and an adjacency heatmap, shown in Fig. 5B and C, respectively. The relationships of the modules with the variables considered are consistent with the correlations shown in Fig. 5A.

**Fig. 6.**
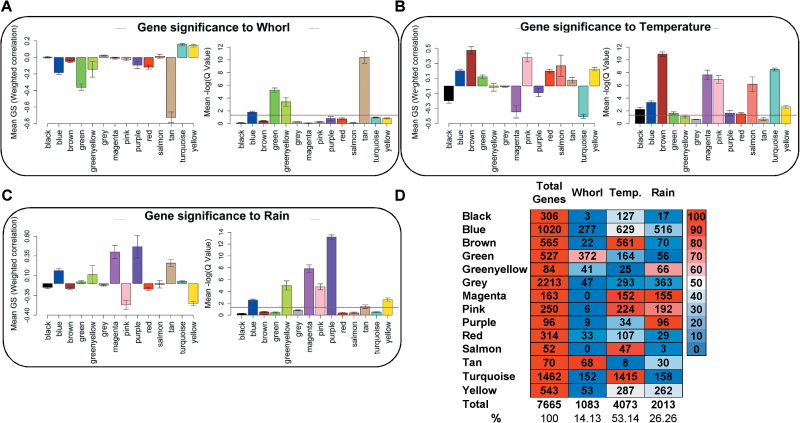
Summary of individual gene-weighted correlations with Whorl, Temperature, and Rain. (A) Means of the weighted correlations between genes and Whorl. (B) Means of the weighted correlations between genes and Temperature. (C) Means of the weighted correlations between genes and Rain. Error bars show standard errors of the samples. Means of the –log10 (*q*-values) of the weighted correlations for each module are shown. The horizontal red line indicates the threshold-significant *q*-value (1.3, the log10 equivalent of 0.05). The bar colours indicate the eigengene modules’ names. (D) Summary of significant weighted correlations (*q*-value < 0.05) between the individual genes and variables. Colours indicate percentages of genes in each eigengene module that have significant correlations with a trait. Intense blue and red indicate low and high numbers of significant correlations, respectively.

Relationships between the variables and the expression of individual genes were established through weighted correlation analysis (Supplementary Data S6). A summary of the correlations with the three main variables (Whorl, Temperature, and Rain) and *q*-values is illustrated in Fig. 6A–C, respectively, and the number of genes with significant correlations within each module is shown in Fig. 6D. They exhibited similar patterns to the module-variable correlations. The tan module had the strongest correlation with Whorl; most of the genes in the module (97%) were significantly correlated with this variable (Pearson’s *r* < –0.7), and it had the most significant mean *q*-value. The brown module had the highest number of significant correlations with Temperature, although only slightly more than several other modules (magenta, pink, salmon, and turquoise). Only four genes in this module had no significant correlations with Temperature. Moreover, more than 90% of the genes in the other four modules had significant correlations with Temperature. The purple module contained genes with the highest individual Rain correlations, but in terms of mean values the magenta module was nearly as strongly correlated with Rain. However, the mean *q*-value was much smaller for the purple module than for the other modules. All of the genes in the purple module had significant correlations with Rain. Of the three main variables, Temperature was significantly correlated with the highest number of genes (4073, 53.14% of the total), followed by Rain (2013, 26.26%) and Whorl (1083, 14.13%).

Ten genes displaying the most significant correlations with each selected variable are listed in Table 2. Genes with the highest correlations to Whorl displayed negative values (<–0.9) and belonged to the tan module. Most of them encode flavonoid metabolism enzymes. The genes most strongly correlated with Temperature also had very strong correlations (> 0.91); all except two (encoding proteins involved in translation: the elongation factor 1-alpha or the initiation factor 4a) belong to the brown module. Finally, the genes most strongly correlated with Rain had correlations higher than 0.91; nine belonged to the purple module and all of them were positively correlated with Rain except for a gene encoding an auxin-responsive protein.

**Table 2.** Genes most correlated with Whorl, Temperature and Rain

Genes most correlated with Whorl					
Array spot	SustainPine3 ID	Description	WGCNA module	q-value	Correlation
Q17L11	sp_v3.0_unigene5895	Armadillo beta-catenin repeat family protein	Tan	< 2.4E–14	–0.92
Q15M17	sp_v3.0_unigene35734	Chalcone synthase	Tan	< 2.4E–14	–0.92
Q18F13	sp_v3.0_unigene17242	Chalcone synthase	Tan	< 2.4E–14	–0.92
Q17O7	sp_v3.0_unigene30516	40S ribosomal protein	Tan	< 2.4E–14	–0.92
Q21C21	sp_v3.0_unigene126627	Flavonoid-3’-5’-hydroxylase	Tan	< 2.4E–14	–0.91
Q15K3	sp_v3.0_unigene19098	Chalcone isomerase	Tan	< 2.4E–14	–0.91
Q13K2	sp_v3.0_unigene25267	Cop9 complex	Tan	< 2.4E–14	–0.91
Q15G15	sp_v3.0_unigene209189	Flavanone 3-hydroxylase	Tan	< 2.4E–14	–0.91
Q16H14	sp_v3.0_unigene16571	Protein-binding protein	Tan	< 2.4E–14	–0.90
Q10C3	sp_v3.0_unigene17073	Geranylgeranyl diphosphate synthase	Tan	< 2.4E–14	–0.90
Genes most correlated with Temperature					
Array spot	SustainPine3 ID	Description	WGCNA module	q-value	Correlation
Q1K1	sp_v3.0_unigene33933	Unknown protein	Brown	<2.221E–16	0.92
Q17J10	sp_v3.0_unigene11216	Ketol-acid reductoisomerase	Brown	<2.221E–16	0.92
Q6B3	sp_v3.0_unigene13258	UDP-arabinose mutase	Brown	<2.221E–16	0.92
Q11E4	sp_v3.0_unigene1012	Elongation factor 1-alpha	Brown	<2.221E–16	0.92
Q22K3	sp_v3.0_unigene18174	Clathrin light chain protein	Brown	<2.221E–16	0.92
Q2A8	sp_v3.0_unigene16921	Eukaryotic initiation factor 4a	Brown	<2.221E–16	0.92
Q16E20	sp_v3.0_unigene142382	3’-5’-Exonuclease domain-containing protein	Brown	<2.221E–16	0.92
Q7G9	sp_v3.0_unigene17036	Triose-phosphate isomerase	Brown	<2.221E–16	0.92
Q17N23	sp_v3.0_unigene4297	Pectin methylesterase-like protein	Pink	<2.221E–16	0.91
Q3A4	sp_v3.0_unigene127113	NA	Turquoise	<2.221E–16	–0.91
Genes most correlated with Rain					
Array spot	SustainPine3 ID	Description	WGCNA module	q-value	Correlation
Q2I24	sp_v3.0_unigene30572	NA	Purple	<1.22E–14	0.94
Q14J11	sp_v3.0_unigene23635	Endoplasmin precursor	Purple	<1.22E–14	0.94
Q5C1	sp_v3.0_unigene37920	Auxin-responsive protein	Purple	<1.22E–14	–0.93
Q15E22	sp_v3.0_unigene7072	Aminoalcoholphosphotransferase 1	Purple	<1.22E–14	0.93
Q19M11	sp_v3.0_unigene7897	3-Hydroxy-3-methylglutaryl synthase	Purple	<1.22E–14	0.92
Q17K21	sp_v3.0_unigene17339	Alcohol dehydrogenase	Purple	<1.22E–14	0.92
Q11J18	sp_v3.0_unigene476	Fatty acid/sphingolipid desaturase	Magenta	<1.22E–14	0.92
Q17J7	sp_v3.0_unigene17840	Heat shock protein 70	Purple	<1.22E–14	0.92
Q11D3	sp_v3.0_unigene18735	Mitochondrial dicarboxylate carrier	Purple	<1.22E–14	0.91
Q12A19	sp_v3.0_unigene34840	Mitochondrial dicarboxylate carrier	Purple	<1.22E–14	0.91

### Validation of expression analysis

The high-throughput expression analysis was validated using RT-qPCR and primers for some of the genes most significantly associated with the variables considered (Table 3). The selected genes showed significant correlations with at least two variables. As shown in Supplementary Figure S7, most of the RT-qPCR results correlate well with the microarray data, displaying no significant deviations except for a few genes.

**Table 3.** Genes used for microarray validation RT-qPCRs and their significant correlations with variables Whorl, Temperature, Rain, and Sucrose

Array spot	SustainPine3 ID	Description	WGCNA module	Whorl	Temperature	Rain	Sucrose
Q7F2	sp_v3.0_unigene5645	CRK1 protein	Blue		Temp.	Rain	Sucrose
Q7N23	sp_v3.0_unigene210278	Myb5	Blue		Temp.	Rain	
Q11C18	sp_v3.0_unigene17872	AP2 ERF domain-containing protein	Red		Temp.		Sucrose
Q11E4	sp_v3.0_unigene1012	Elongation factor 1-alpha	Brown		Temp.		Sucrose
Q13B15	sp_v3.0_unigene599	Hexose transporter	Magenta		Temp.		Sucrose
Q13C16	sp_v3.0_unigene17589	NA	Turquoise		Temp.		Sucrose
Q14O21	sp_v3.0_unigene5704	Subtilase family protein	Red	Whorl	Temp.		Sucrose
Q15M17	sp_v3.0_unigene35734	Chalcone synthase	Tan	Whorl		Rain	
Q16J12	sp_v3.0_unigene7250	Aspartic proteinase nepenthesin I	Red				Sucrose
Q21C21	sp_v3.0_unigene126627	Flavonoid-3’-5’-hydroxylase	Tan	Whorl			

### Functional gene-enrichment analyses

**Fig. 7.**
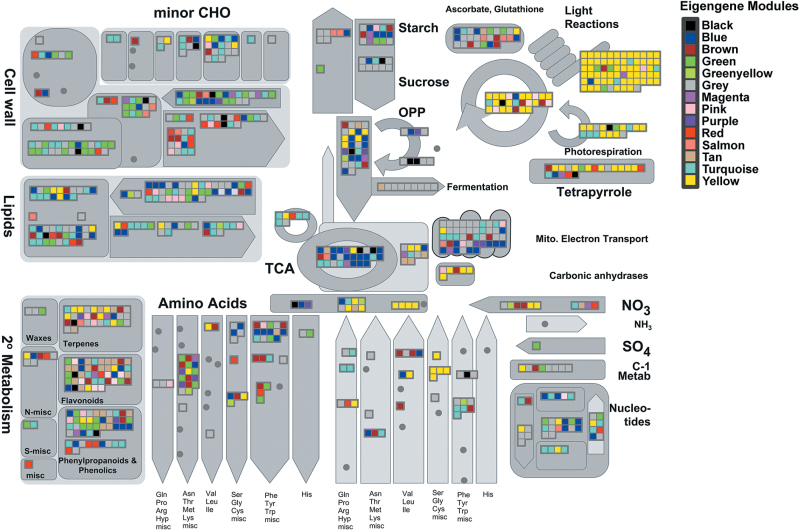
Overview of metabolism in the needles, indicating metabolic functions of the eigengene modules. The Mapman image shows metabolic functions assigned to genes in each module. Colours in the boxes indicate specific functions assigned to specific modules.

The biological significance of the gene co-expression modules was analysed by SEA of the GO terms assigned to genes in each module. The entire results of the SEA are shown in Supplementary Data S7. The GO terms for biological processes found to be enriched in each module are presented in Supplementary Data S7 (no data for the green-yellow, magenta, red, and salmon modules are presented since no significant enrichment of these terms was detected in them). A Mapman visualization of the metabolic functions represented in each module is presented in Fig. 7. The module with the most clearly defined function is the yellow module, which consists of genes involved in metabolic processes related to light and photosynthesis.

The blue, brown, turquoise, and yellow modules are all involved in protein metabolism (ribosomes, translation, transport, and degradation for blue; degradation and transport for brown; synthesis for yellow; and transport for turquoise). Moreover, the blue, brown, and yellow modules are also related to carbon metabolism (lignin, sterol, and fatty acid biosynthesis for blue; carbohydrate and lipid metabolism for brown; and glycolysis, monocarboxylic acid, and carboxylic acid catabolism for yellow). The blue module also seems to be involved in responses to external stimuli (cold, heat, salt, cadmium, and reactive oxygen species) and energy metabolism (ATP synthesis coupled to proton transport). The turquoise module shows enrichment in cell development and intracellular transport terms, while the black module is related to development and Golgi vesicle transport. The pink and purple modules are related to processes that are more clearly defined in the purple module (responses to oxidative, cold, and osmotic stresses and hormone stimuli). Finally, the green and tan modules showed overrepresentation of genes of the flavonoid biosynthesis pathway and (in the green module) functions associated with flavonoids (e.g. light and wounding responses), lignin and cell wall biosynthesis, cell growth, and responses to cold stress and water deprivation

The tan group was the only module with no significant Temperature correlation, but it was highly correlated with Whorl. It contains a relatively low number of genes but includes genes encoding the main enzymes of the KEGG flavonoid biosynthesis pathway (ko00941), and together with the green module provides most of this pathway (Supplementary Figure S8). These two modules also provide nearly all of the key enzymes of the shikimate/phenylalanine (ko00400) and phenylpropanoid (ko00940) pathways (Supplementary Figure S8). Placement of the two modules’ genes in the KEGG biosynthesis of secondary metabolites map (ko01110) also showed that the terpenoid backbone pathway is represented in them (Supplementary Figure S9). The green and tan modules also include genes for transcription factors, suggesting they may be involved in regulation of the phenylpropanoid, flavonoid, and terpenoid biosynthetic pathways. Good candidates in the green and tan modules were genes encoding the R2R3-Myb transcription factors Q11F13 and Q16P17, since members of the R2R3-Myb family regulate these metabolic pathways in conifers (Bedon *et al.*, 2010).

## Discussion

### Metabolite profiles

In this work we examined both seasonal and developmental changes in metabolite and transcriptome profiles in needles of adult maritime pine trees growing in natural conditions. The results reflect the complex interactions between developmental processes and environmental adaptations of conifer trees in nature. The metabolite profiles of the needles mainly changed with developmental stage, and thus the whorl in which they were located, from the youngest to the oldest (Fig. 2, Supplementary Figure S4). However, the accumulation pattern of sucrose and some other metabolites seems also to be affected by the environmental conditions. Therefore, developmental time appears to be a long-term variable that strongly influences metabolite levels, suggesting that metabolic processes are similar in all whorls.

Levels of an important group of metabolites, mainly involved in C1 and redox metabolism, seem to decrease with increases in needle age, most clearly in the oldest, third whorl (Fig. 2, Table 1). One of these metabolites, betaine, has been intensively studied in herbaceous plants due to the importance of its roles as a ‘compatible solute’ in resistance to abiotic stresses such as salinity and photoinhibition (Chen and Murata, 2011). Little is known about the roles of betaine in conifers, but it seems to be related to *S*-adenosyl-methionine metabolism (Mayne *et al.*, 1996). This possibility is nicely supported by the observed relationship between betaine and methionine levels (Fig. 2). Moreover, our results suggest the involvement of these two metabolites in methyl-THF metabolism. We have found strong correlations between these particular metabolites and genes encoding methylenetetrahydrofolate dehydrogenase (Q10D23; MTHFD, EC 1.5.1.5) and serine hydroxymethyltransferase (Q1E13; SHMT, EC 2.1.2.1) (Supplementary Data S8). All these results suggest that both metabolites could be involved in the formation of the cell wall through metabolism of the methyl-THF. Accordingly, SHMT activity is essential for methyl-THF regeneration during lignification in maritime pine (Villalobos *et al.*, 2012). Betaine could also be involved in protecting cells in the younger needles from various stresses. The observed reductions in the levels of methionine-related metabolites in the older needles also suggest a role in photosynthesis or responses to oxidative stress consistent with reported reductions in photosynthetic rates in older needles of *P. pinaster* (Warren, 2005).

In contrast, carbon metabolism seems to be affected by long-term factors that increase levels of the main carbon metabolites as whorls age. This may be because young whorls have high metabolic rates and hence highly dynamic carbohydrate catabolism while, in contrast, old whorls may assimilate surplus carbon and play a role as metabolite stores. This is consistent with two of the strongest correlations detected, between the expression of a gene encoding a hexose transporter and the relative abundances of sucrose and pinitol (Supplementary Data S8). The putative storage role is also supported by reported reductions in photosynthetic rates with ageing in *P. pinaster* needles (Warren, 2005). Furthermore, Rubisco activity is downregulated during aging, in accordance with its postulated role as a nitrogen store (Gezelius and Hallén 1980, Näsholm and Ericsson, 1990; Stitt and Schulze, 1994; Warren, 2005; Galindo-González *et al.*, 2012). The observed profiles of sugars and methionine-related metabolites also suggest they play coordinated metabolic roles during ageing of maritime pine needles. Notably, pinitol is a ‘compatible solute’ involved in tolerance to osmotic and oxidative stresses in plants (Simard *et al.*, 2013), and sucrose has cryoprotectant and osmoprotectant functions during overwintering (Galindo-González *et al.*, 2012). In this sense, the accumulation patterns of sucrose and some other metabolites also reflect potential roles in the winter acclimation or in the responses to the environment or physiological status.

### Transcript profiles

Our data indicate that developmental stage is the main determinant of metabolite levels in maritime pine needles, but temperature is the main variable influencing their transcriptome profiles (Fig. 1, 4–7). The abundance of transcripts increased in the warmer months, particularly transcripts involved in photosynthesis (yellow module) and carbohydrate metabolism (yellow, blue, and brown modules) (Fig. 4, Supplementary Data S7). These findings are consistent with well established seasonal shifts in rates of photosynthesis in response to changes in temperature (Medlyn *et al.*, 2002; Öquist and Huner, 2003; Ensminger *et al.*, 2004; Gea-Izquierdo *et al.*, 2010). In addition, there were coordinated shifts in metabolic pathways related to photosynthesis. Notably, increases in temperature induced increases in transcripts of genes encoding enzymes involved in photorespiration and nitrogen metabolism included in the yellow module, such as alanine-glyoxylate aminotransferase (*AGT*, EC 2.6.1.44), serine hydroxymethyltransferase (*SHMT*, EC 2.1.2.1), and the P-protein of the glycine decarboxylase complex (*GLDP*, EC 1.4.4.2) (Fig. 7, Supplementary Data S7). These data corroborate findings we have previously reported regarding the differential regulation of *GS* genes. For example, GS1a (EC 6.3.1.2), a cytosolic GS responsible for reassimilation of ammonium released during photorespiration in photosynthetic cells (Cantón *et al.*, 1999; Cánovas *et al.*, 2007) was present in the yellow module, together with genes involved in photosynthesis and photorespiration. The results reported here also support the key role of GS1b in amino acid and secondary metabolism (Avila *et al.*, 2001; Cánovas et al, 2007; Craven-Bartle *et al.*, 2013) as part of the brown module, which is enriched in genes associated with aromatic amino acid biosynthesis (Fig. 7, Supplementary Data S7). Even though the changes in the transcript profiles were well correlated with the temperature, other factors may influence gene expression. For example, the photoperiod can induce changes in the expression of certain genes assigned to temperature. However, it is very difficult to distinguish between photoperiod- or temperature-induced effects due to their parallel evolution during the year (Supplementary Figure S2). It is well documented that photoperiod and temperature have interconnected influence over physiological and metabolic processes in plants (Öquist and Huner 2003; Verhoeven, 2014).

Interestingly, the expression of an important group of genes in the turquoise module increased under low temperature (Fig. 4, Supplementary Data S7). These genes may participate in the maintenance of basal cellular functions when photosynthetic rates are low, or in general adaptations to cold conditions, as previously reported in stems of *P. glauca* during preparation for overwintering (Galindo-González *et al.*, 2012) or in *P. sitchensis* (Grene *et al.*, 2012; Collakova *et al.*, 2013). Upregulation of glycolysis, the TCA cycle, oxidative phosphorylation, and other energy-generating pathways during winter is essential for plants to survive the reductions in photosynthetic generation of ATP (Ophir *et al.*, 2009; Grene *et al.*, 2012, Galindo-González *et al.*, 2012; Collakova *et al.*, 2013). Functional enrichment in the turquoise module suggests that it is involved in associated metabolic reorganization during the winter (Supplementary Data S7), including adaptation of carbohydrate metabolism and oxidative phosphorylation (Supplementary Data S7 and Fig. 7). The module may also participate in shifts in lipid metabolism (Fig. 7) linked to observed changes in the lipid composition of cellular membranes in response to cold conditions in pines (Roden *et al.*, 2009).

The purple module correlated most strongly with rainfall, and all genes in the module correlated significantly with rainfall (Fig. 5 and 7), suggesting that it is heavily involved in responses to shifts in water status and availability. The module also included several genes related to drought stress, such as one encoding an auxin-responsive protein (Q1J15). Furthermore, it was functionally enriched in oxidative stress-response genes, which could have been partially due to the coincidence of rainfall with low temperature periods during our study period (Supplementary Data S7). However, winter cold and desiccation stresses or over-excitation of photosystem II can result in oxidative stress (Öquist and Huner, 2003), providing another possible reason for the linkage. We also detected a gene encoding a cold acclimation protein in the module (Q12L10) and genes involved in lipid metabolism, as in the turquoise module (Supplementary Data S5).

The gene expression profiles in the needles of Whorl 0 reflect early developmental phases, including very significant changes during early leaf expansion stages (May and July). In *P. pinaster*, leaf expansion seems to be completed in several months, from spring to autumn (Warren, 2005), but in some modules gene expression differences persisted for longer (Fig. 4). The tan module (the only module with no significant correlation with Temperature) was the most strongly correlated with Whorl. It was particularly enriched in flavonoid biosynthesis genes, as possibly reflected in the light-green colour of the needles of Whorl 0, in marked contrast to the deep green (almost blue) colouration of older needles (Fig. 1C). The biosynthesis of flavonoids and terpenoids may be related to UV and freezing stress protection (Teklemariam and Blake, 2004; Jaakola and Hohtola, 2010). Comparison of the green and tan modules showed that they contain genes with closely related functions, including participation in the shikimate, phenylpropanoid, and flavonoid synthesis pathways (Supplementary Figures S4 and S5). This suggests that they play important roles in cell wall synthesis and coordinated processes during the expansion of maritime pine needles. Moreover, the division of these genes into two modules and the presence of different isogenes encoding proteins with the same function (Supplementary Figure S8) suggest that several gene packages participate in the same metabolic pathways, possibly providing essential flexibility in responses to varying environmental and/or developmental conditions.

### Concluding remarks

We have identified a number of gene co-expression networks in the needles of maritime pine trees, confirming that such analysis can provide valuable indications of the functions of gene clusters and their co-regulation (Basnet *et al.*, 2013; Hirano *et al.*, 2013). Unravelling these networks is highly important for understanding conifer tree development and adaptation to environmental stimuli. For instance, we have identified candidate regulatory genes such as Myb transcription factors (Q11F13 and Q16P17) that may participate in regulation of phenylpropanoid and flavonoid biosynthesis.

We cannot be sure that the responses to environmental conditions of this pine population in southern Spain are representative of other pine populations. The expression of specific genes and accumulation of metabolites related to environmental adaption could be different in other contexts including the same maritime pine population growing at seaside levels, as suggested by the phenological timing differences.

**Fig. 8.**
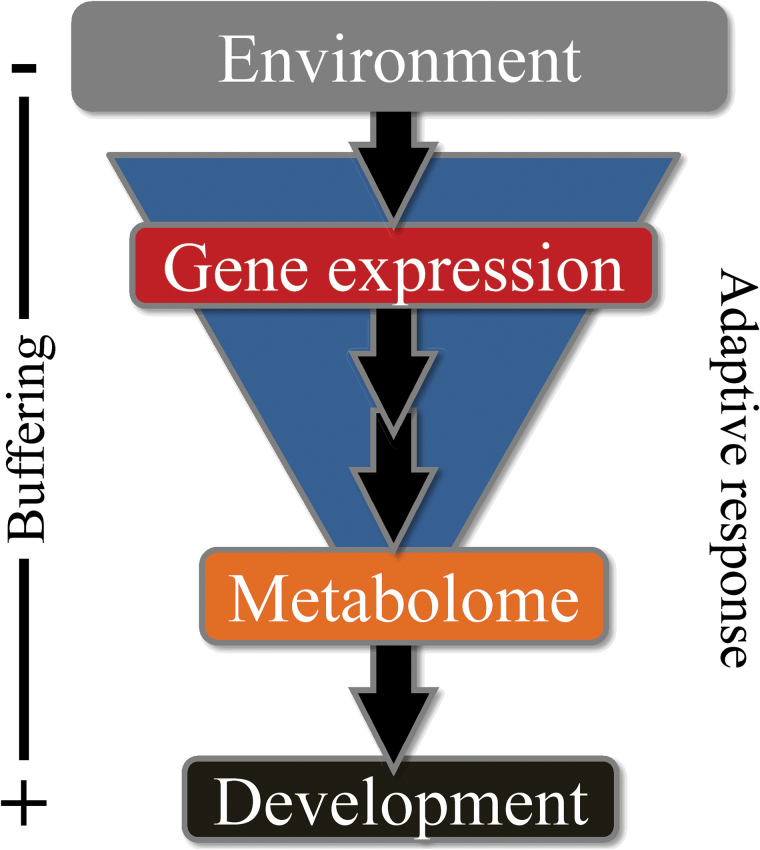
Proposed model for adaptive responses in maritime pine. This figure is available in colour at *JXB* online.

In summary, our results strongly suggest that environmental changes modulate the transcriptome for fine regulation of the metabolome during development (Fig. 8). According to this model, adaptive responses in maritime pine would influence the developmental programme through the maintenance of a metabolic homeostasis. In this sense our results strongly suggest the existence of alternative gene packages involved in the same metabolic pathways contributing to essential flexibility in responses to varying environmental and/or developmental conditions. The system-based analysis presented here provides a general vision of the seasonal regulation of maritime pine growth and opens new perspectives for understanding the complex regulatory mechanisms underlying adaptive responses in conifers. In ongoing work we are extending the functional characterization of key regulatory factors identified here, and others involved in controlling conifer tree development and environmental adaptation.

## Supplementary material

Supplementary data can be found at *JXB* online.

Supplementary Table S1. Sampling dates.

Supplementary Table S2. Primers used for RT-qPCR.

Supplementary Figure S1. Microarray hybridization strategy.

Supplementary Figure S2. Annual photoperiod, average monthly temperature, and monthly accumulated rainfall; and Pearson correlations between eigengene modules and environmental and developmental factors.

Supplementary Figure S3. Average monthly temperature since 2007 and monthly temperature in 2012; and average monthly accumulated rainfall since 1994 and monthly accumulated rainfall in 2012.

Supplementary Figure S4. Metabolite-weighted co-expression network heatmap and correlations between eigengene modules and traits.

Supplementary Figure S5. Global network representation including both a global network heatmap plot and a multidimensional scaling plot from WGCNA.

Supplementary Figure S6. Normalized expression values for each gene in the gene co-expression modules from WGCNA.

Supplementary Figure S7. Comparison between RT-qPCR and microarray expression data to validate the microarray hybridizations.

Supplementary Figure S8. KEGG shikimate, phenylalanine, phenylpropanoid, and flavonoid pathways with functional assignments for the green and tan eigengene modules.

Supplementary Figure S9. KEGG pathway for the biosynthesis of secondary metabolites (ko01110) including the brown and green-yellow modules.

Supplementary Method S1. Metabolite network analysis (WGCNA) script.

Supplementary Method S2. Metabolite PCA R script.

Supplementary Method S3. BABAR script for microarray data normalization.

Supplementary Method S4. Gene network analysis (WGCNA) script.

Supplementary Data S1. Metabolites in the spectra library and normalized metabolite contents.

Supplementary Data S2. Removed gene list due to missing values.

Supplementary Data S3. Connectivity (kME) and module membership (MM) values for each gene.

Supplementary Data S4. Weighted correlations between variables (Whorl, Temperature, and Rain) and individual profiles of metabolite amounts.

Supplementary Data S5. Normalized gene expression and eigengene module assignment.

Supplementary Data S6. Weighted correlations between traits (Whorl, Temperature. Rain, and Sucrose) and individual gene expression.

Supplementary Data S7. SEA of the GO terms assigned to genes in each module.

Supplementary Data S8. Weighted correlations between metabolites and individual gene expression.

## Funding

This work was supported by the grants from the European Commission’s 7th Framework Programme (FP7-KBBE- 2011–5) and the Spanish Ministerio de Economía y Competitividad grant (BIO2012-33797).

## Supplementary Material

Supplementary Data
