# Supplementary material for: Understanding developmental and adaptive cues in pine through metabolite profiling and co-expression network analysis
Source: J Exp Bot. 2015 Apr 1;66(11):3113–27. doi: 10.1093/jxb/erv118 (PMC4449534; doi:10.1093/jxb/erv118)
Supplement: Supplementary Data [file supp_66_11_3113__index.html]

Understanding developmental and adaptive cues in pine through metabolite profiling and co-expression network analysis — Understanding developmental and adaptive cues in pine through metabolite profiling and co-expression network analysis — Supplementary Data 

# Understanding developmental and adaptive cues in pine through metabolite profiling and co-expression network analysis

## Supplementary Data

Data files

**Files in this Data Supplement:**

- Supplementary Data - Supplementary Data
