# Supplementary material for: Understanding developmental and adaptive cues in pine through metabolite profiling and co-expression network analysis
Source: J Exp Bot. 2015 Apr 1;66(11):3113–27. doi: 10.1093/jxb/erv118 (PMC4449534; doi:10.1093/jxb/erv118)
Supplement: Supplementary Data [file supp_erv118_jexbot142638_file001.pdf]

Table S1. Sampling dates.

Month	Sampling date	Temp. at sampling hour	NMR	Microarray
January	1/30/2012	6.6 ºC	Yes	Yes
February	2/29/2012	11.2 ºC	Yes	
March	3/30/2012	5.7 ºC	Yes	Yes
April	4/26/2012	13.3 ºC	Yes	
May	5/30/2012	16.6 ºC	Yes	Yes
June	6/26/2012	27.0 ºC	Yes	
July	7/27/2012	16.5 ºC	Yes	Yes
August	8/28/2012	26.1 ºC	Yes	
September	9/28/2012	8.2 ºC	Yes	Yes
October	10/30/2012	9.0 ºC	Yes	
November	11/30/2012	0.5 ºC	Yes	Yes
December	12/27/2012	9.8 ºC	Yes	

Table S2. List of primers used for RT-qPCR.

Primer	Primer sequence	SustainPine ID	Description
qSP3-18113-F	ATCTCTCAGCACATTCCAACAG	sp_v3.0_unigene18113	Actin
qSP3-18113-R	TATTGCCACCATCATCTCAAGC	sp_v3.0_unigene18113	Actin
qSP3-4304-F	CGGTTGTTGTATGTGGGAGC	sp_v3.0_unigene4304	40S Ribosomal protein S27
qSP3-4304-R	AGCCTTCAGTTAGTCGTGCT	sp_v3.0_unigene4304	40S Ribosomal protein S27
qSP3-1012-F	TGCTGTTGGAGTCATCAAGG	sp_v3.0_unigene1012	Elongation factor 1-alpha
qSP3-1012-R	CATTTACCCTTCTTGGCCGC	sp_v3.0_unigene1012	Elongation factor 1-alpha
qSP3-21410-F	CCATTGCAGTCTGTGCATCT	sp_v3.0_unigene21410	Tubuline 8
qSP3-21410-R	GCACAGCAATATGGATCTGGT	sp_v3.0_unigene21410	Tubuline 8
qSP3-96846-F	TTGGCTGTTGTGTGTCAATG	sp_v3.0_unigene96846	Ubiquitin
qSP3-96846-R	ACCGACCGACAAACCAAGTA	sp_v3.0_unigene96846	Ubiquitin
qSP3-35734-F	GATCGCCTAGCAAATAAATCGC	sp_v3.0_unigene35734	Chalcone synthase
qSP3-35734-R	TGTGTTTCTGTCTGGTTCTGG	sp_v3.0_unigene35734	Chalcone synthase
qSP3-126627-F	TCTCTACTAATCACAGACGCAGC	sp_v3.0_unigene126627	Flavonoid-3'-5' -hydroxylase
qSP3-126627-R	TGTGCATAAACTTTGCCTCTACG	sp_v3.0_unigene126627	Flavonoid-3'-5' -hydroxylase
qSP3-210278-F	TTCTCACATGGGGTTGCAAGT	sp_v3.0_unigene210278	Myb5
qSP3-210278-R	GACACCGGAAGCACTCTCTT	sp_v3.0_unigene210278	Myb5
qSP3-5645-F	TCAGGGGTTTTCATTCTATAGCA	sp_v3.0_unigene5645	CRK1 protein
qSP3-5645-R	GTTGCCAACACTTATTGCAGTT	sp_v3.0_unigene5645	CRK1 protein
qSP3-17872-F	GTCTAGTGGCCAGCATGAAATT	sp_v3.0_unigene17872	AP2 ERF domain-containing protein
qSP3-17872-R	GGCCTCACATAAATTGCACCT	sp_v3.0_unigene17872	AP2 ERF domain-containing protein
qSP3-7250-F	TCATCTGCACCGTCTACTTCT	sp_v3.0_unigene7250	Aspartic proteinase nepenthesin I
qSP3-7250-R	ACATTTCATAAGACCTAGCAGCA	sp_v3.0_unigene7250	Aspartic proteinase nepenthesin I
qSP3-5704-F	GCTTTTGGTCCTGGAACTTCTG	sp_v3.0_unigene5704	Subtilase family protein
qSP3-5704-R	GCTTGCCCCTTCACTTCAAC	sp_v3.0_unigene5704	Subtilase family protein
qSP3-17589-F	AGATGCCTAATGTTGTGCAGT	sp_v3.0_unigene17589	NA
qSP3-17589-R	ACATCTAACAAGTTGCCGGAC	sp_v3.0_unigene17589	NA
qSP3-599-F	ACAGCGAGAATCACAGATCCA	sp_v3.0_unigene599	Hexose transporter
qSP3-599-R	CTTGCGAGTAATAAGCGGTAGT	sp_v3.0_unigene599	Hexose transporter
qSP3-17073-F	AAGACAAAACTGAGAAGCTTGC	sp_v3.0_unigene17073	geranylgeranyl diphosphate synthase
qSP3-17073-R	GATGTCTGAGCAAGTGGGAA	sp_v3.0_unigene17074	geranylgeranyl diphosphate synthase
qSP3-27138-F	CTGCAATGTCATGGGAAGCA	sp_v3.0_unigene27138	Myb23
qSP3-27138-R	AGGAATTTAATCATCCTGCACCT	sp_v3.0_unigene27138	Myb23
qSP3-33248-F	ATTCTAATTCTGGCCACGGG	sp_v3.0_unigene33248	Myb20
qSP3-33248-R	GCTGCGATAATCCACACCCT	sp_v3.0_unigene33248	Myb20
qSP3-6029-F	GCTCCTCTTAAGCGTGCTAA	sp_v3.0_unigene6029	arogenate prephenate dehydratase
qSP3-6029-R	GAAGTATAGTCGCGCACACC	sp_v3.0_unigene6029	arogenate prephenate dehydratase
qSP3-8540-F	GCAGACTGGCAGCAAAATGA	sp_v3.0_unigene8540	4-coumarate: ligase
qSP3-8540-R	CGCTTACTCTGCACCACTTT	sp_v3.0_unigene8540	4-coumarate: ligase
qSP3-31154-F	CCACCTCCACCGTTGAAATC	sp_v3.0_unigene31154	4-coumarate: ligase
qSP3-31154-R	GTATTGGCCGCCGTCATG	sp_v3.0_unigene31154	4-coumarate: ligase
qSP3-16566-F	AGATTGCAAGCCATGGAGGG	sp_v3.0_unigene16566	prephenate aminotransferase
qSP3-16566-R	GCATGTCATCATTGCCGAAGGC	sp_v3.0_unigene16566	prephenate aminotransferase

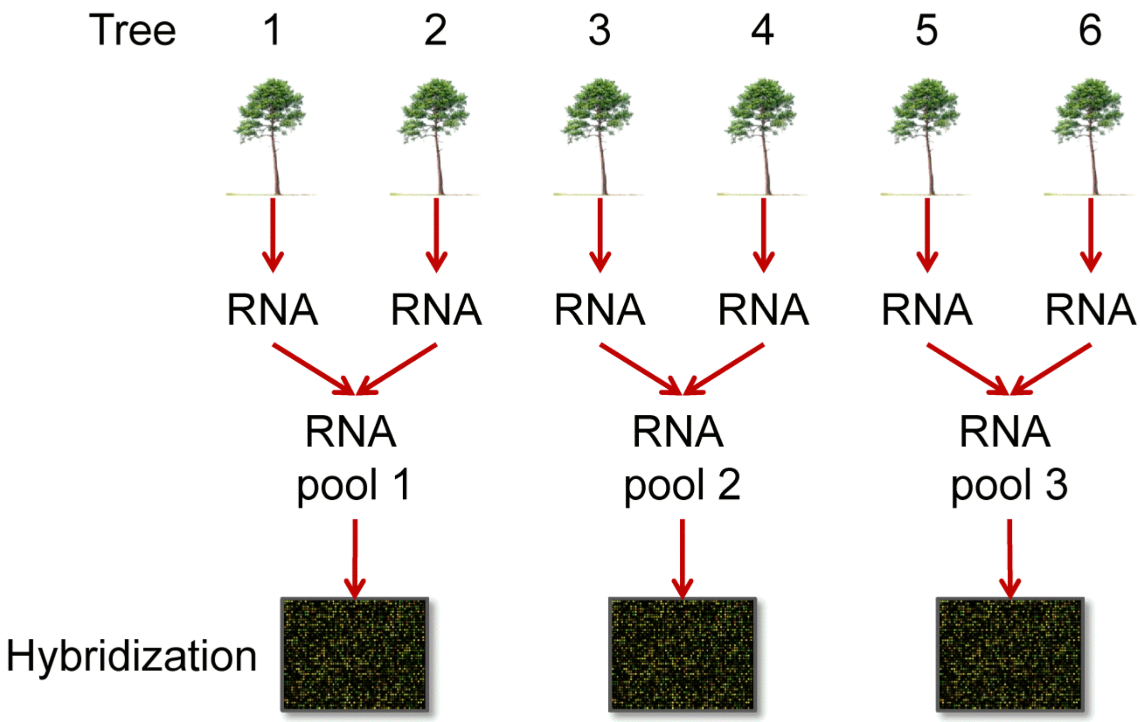

Figure S1. Microarray hybridization strategy.

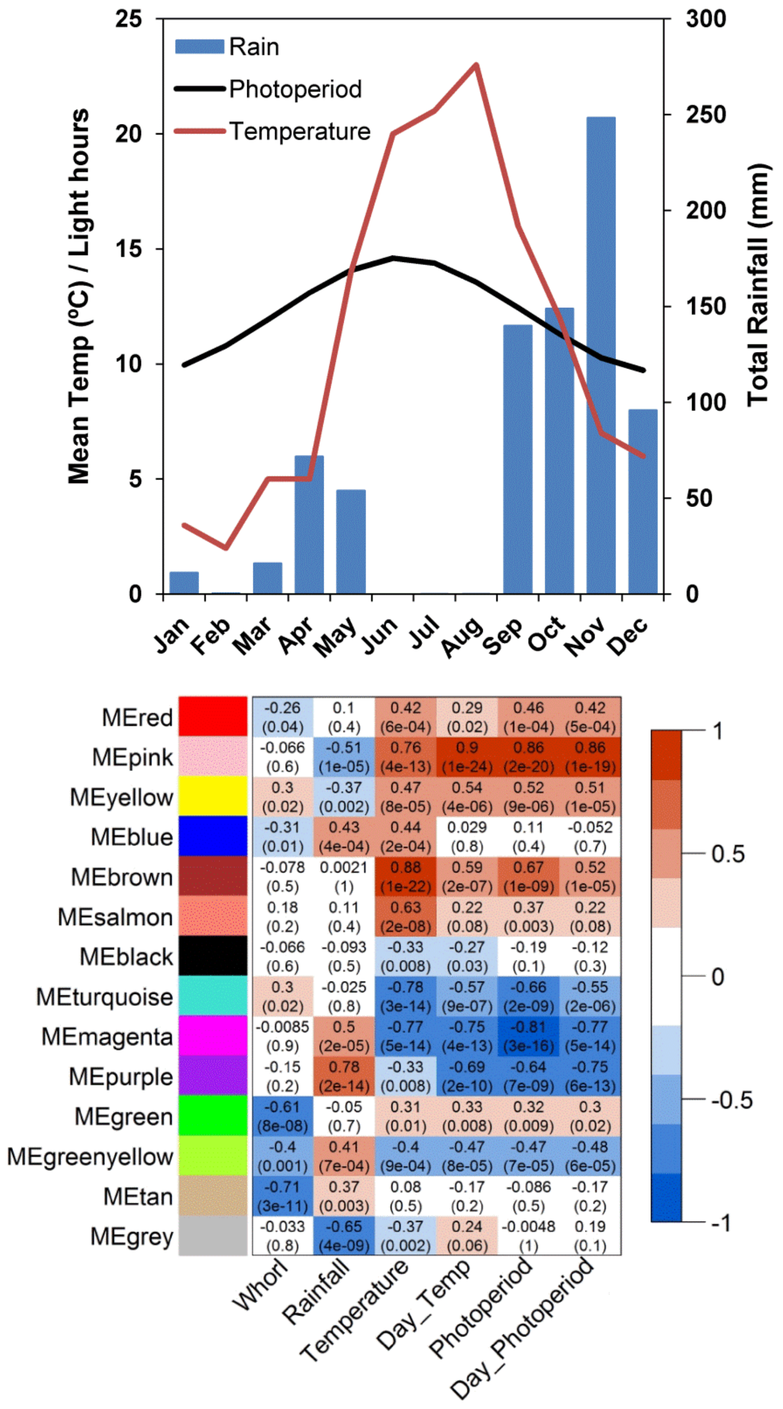

Figure S2. Upper panel. Annual photoperiod, average monthly temperature and monthly accumulated rainfall. **Lower panel.** Pearson correlations between eigengene modules and environmental and developmental factors, including temperature at sample harvesting hour and photoperiod at sampling day.

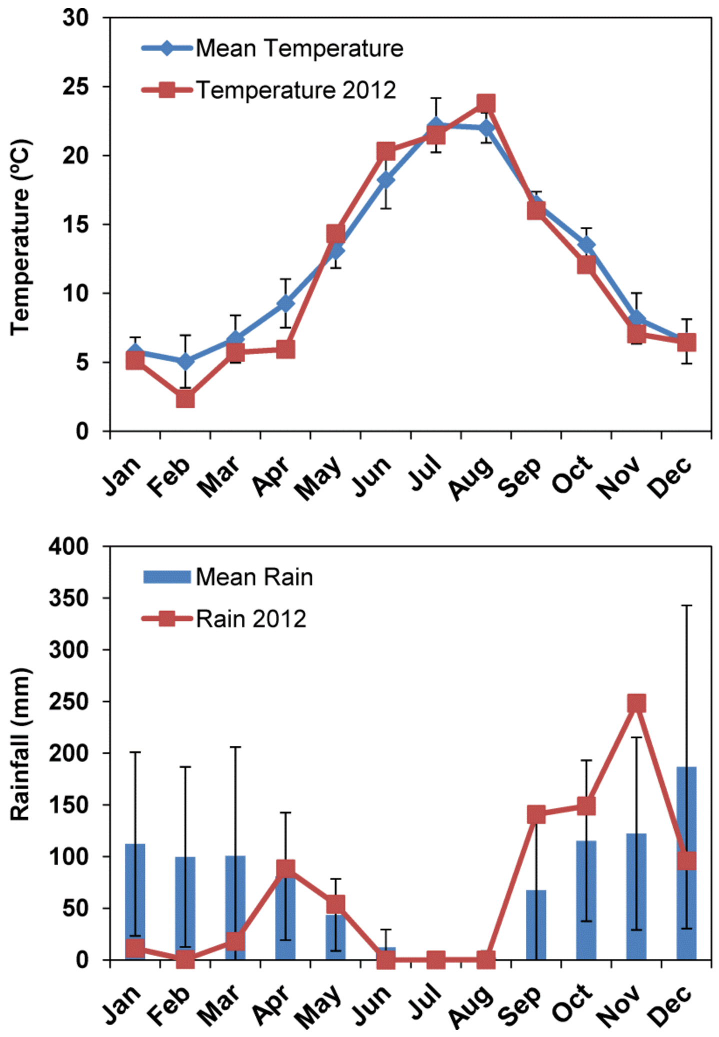

Figure S3. Upper panel. Average monthly temperature since 2007 and monthly temperature in 2012. **Lower panel.** Average monthly accumulated rainfall since 1994 and monthly accumulated rainfall in 2012.

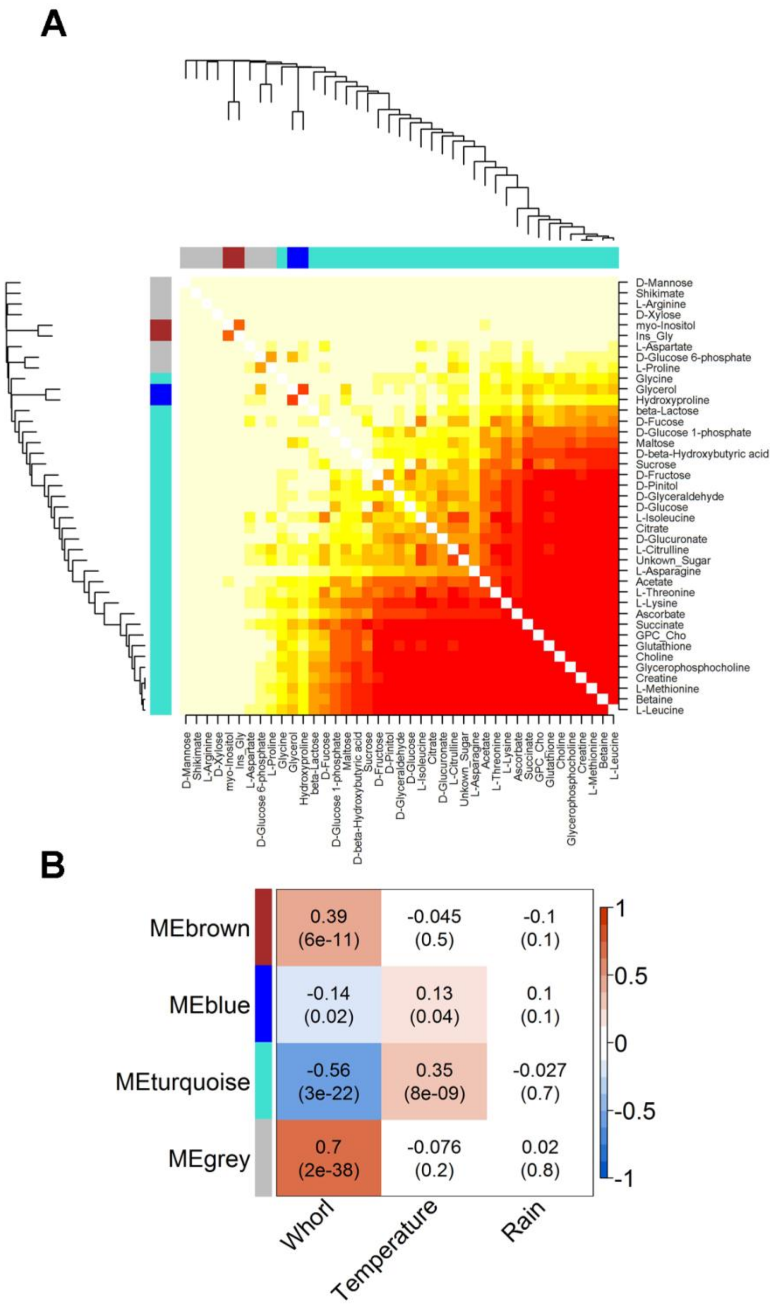

Figure S4. Metabolite-weighted co-expression network heatmap and correlations between eigengene modules and traits. A Metabolite network heatmap and dendrogram obtained with WGCNA. The intensity of red coloration indicates the strength of relationships between metabolites. **B** Pearson correlations between metabolite levels and Whorl, Temperature and Rain, with *p-values* in parenthesis. The correlations are deemed to be significant when $p < 0.05$. The colours in the matrix boxes show the correlation magnitude and direction; intense blue and red indicate strong negative and positive correlations, respectively. High temperature is positively correlated with the metabolite accumulation. Older whorls are positively correlated with metabolite accumulation

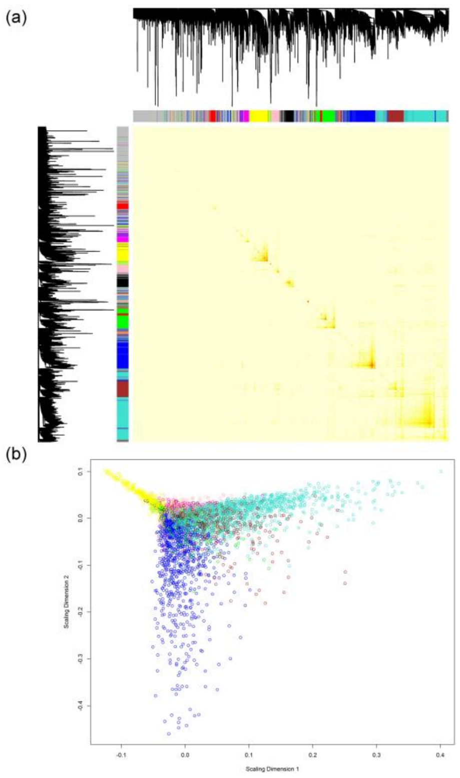

Figure S5. Global network representation. (a) Global network heatmap plot from WGCNA. (b) Multidimensional scaling (MDS) plot from WGCNA.

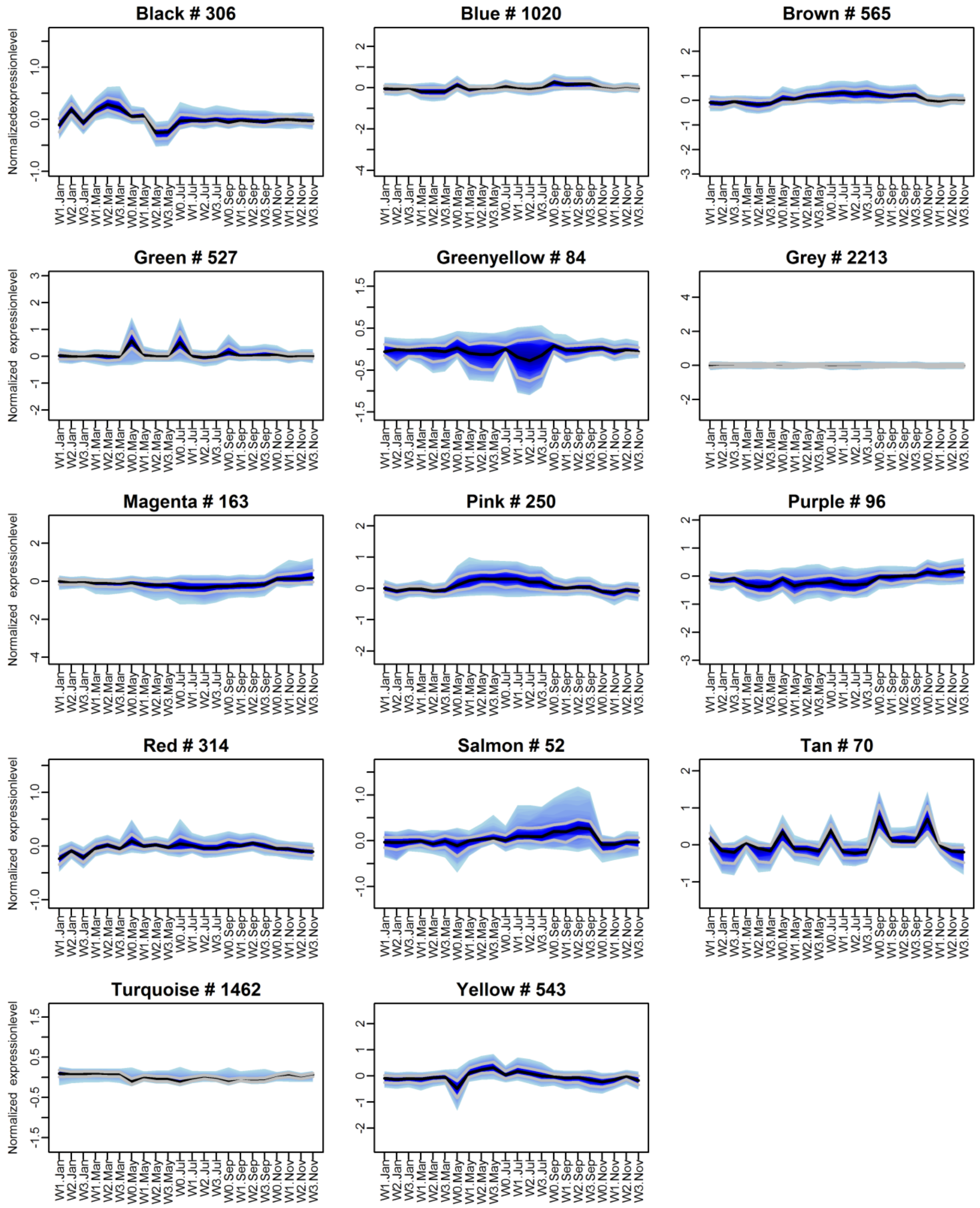

Figure S6. Normalized expression values for each gene in the gene co-expression modules from weighted gene co-expression network analysis (WGCNA). The number of genes in each group is indicated close to the module name. Quartiles are indicated by grey lines for 0.25 and 0.75 and a black line for 0.5.

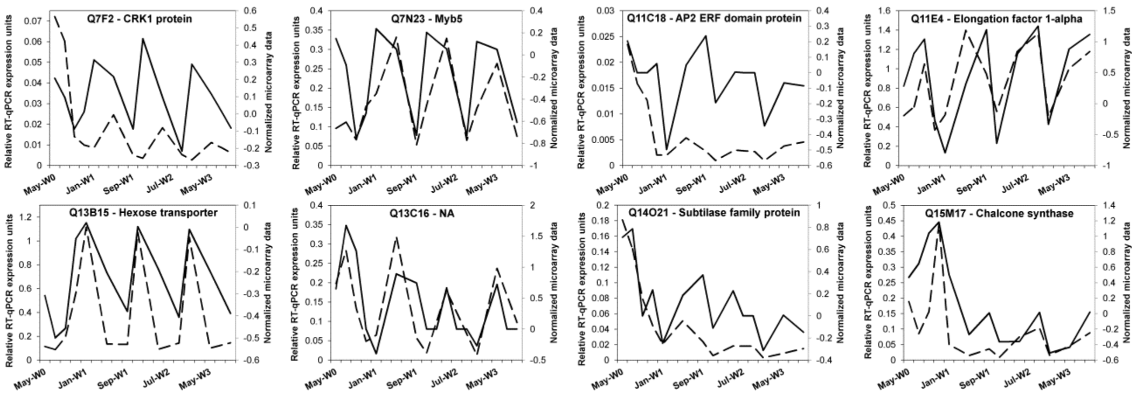

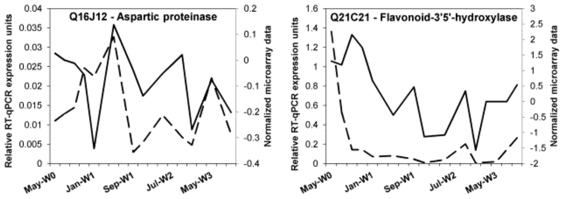

Array spot	Sustainpine ID	Description	Correlation	*p-value*
Q7F2	sp_v3.0_unigene5645	CRK1 protein	**0.04**	**0.82**
Q7N23	sp_v3.0_unigene210278	Myb5	0.50	1.88E-03
Q11C18	sp_v3.0_unigene17872	AP2-ERF domain protein	0.56	0.01
Q11E4	sp_v3.0_unigene1012	Elongation factor 1-alpha	0.68	2.23E-06
Q13B15	sp_v3.0_unigene599	Hexose transporter	0.71	3.66E-07
Q13C16	sp_v3.0_unigene17589	Unknown protein	0.51	0.02
Q14O21	sp_v3.0_unigene5704	Subtilase family protein	0.88	3.77E-08
Q15M17	sp_v3.0_unigene35734	Chalcone synthase	0.74	5.78E-08
Q16J12	sp_v3.0_unigene7250	Aspartic proteinase	**0.32**	**0.07**
Q21C21	sp_v3.0_unigene126627	Flavonoid-3'5'-hydroxylase	**0.33**	**0.05**

Figure S7. Comparison between RT-qPCR and microarray expression data to validate the microarray hybridizations. The continuous line corresponds to the microarray data and the broken line to the RT-qPCR data. Pearson correlations between both pool of data for each gene and their *p-values* are shown in the table. The correlations are significant with a *p-value* < 0.05.

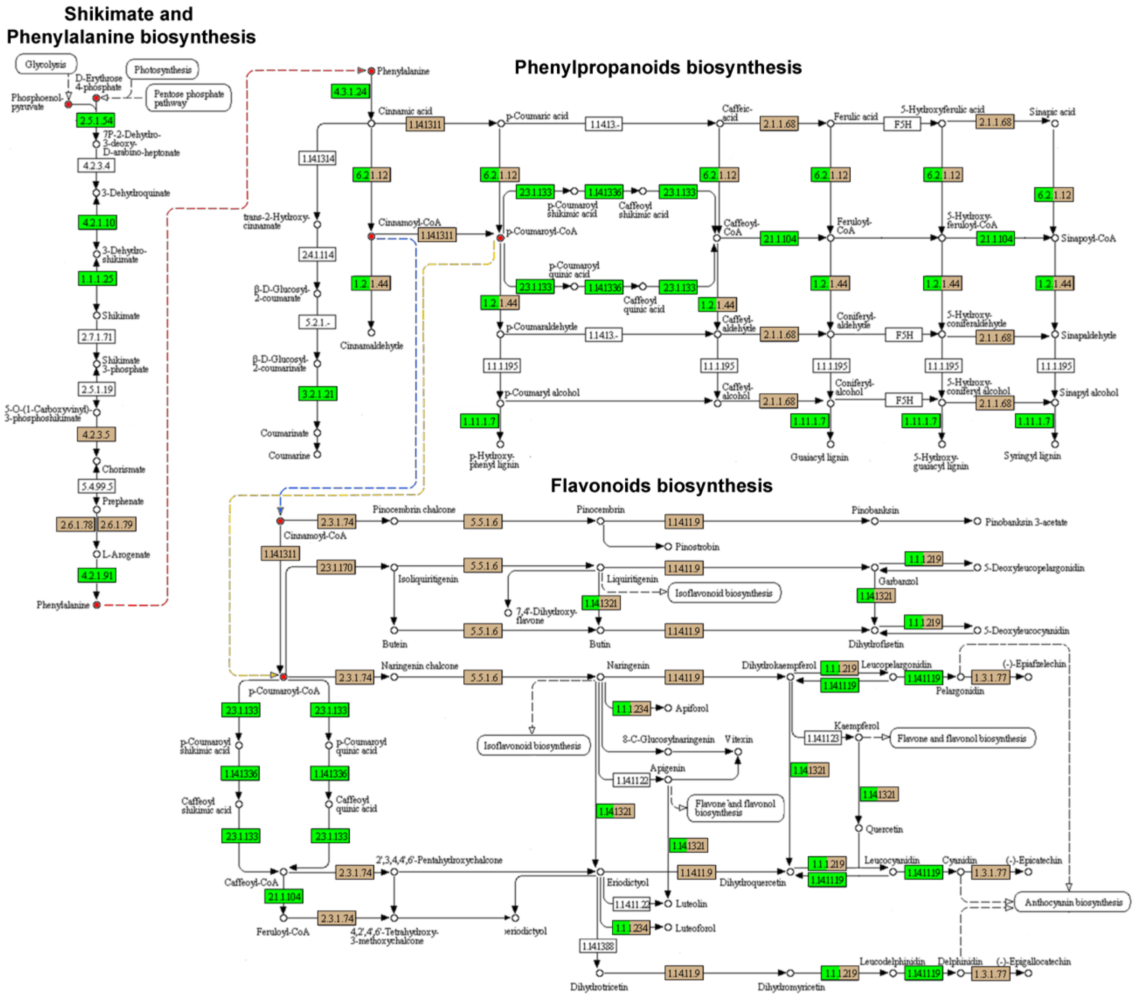

Figure S8. KEGG shikimate, phenylalanine, phenylpropanoid and flavonoid pathways with functional assignments for the green and tan eigengene modules. Functions assigned to genes of the green and tan eigengene modules are indicated by the green and tan colours in the reaction boxes, respectively. The id numbers for the original KEGG pathways were: ko00400 (shikimate and phenylalanine biosynthesis), ko00940 (phenylpropanoid biosynthesis) and ko00941 (flavonoid biosynthesis).

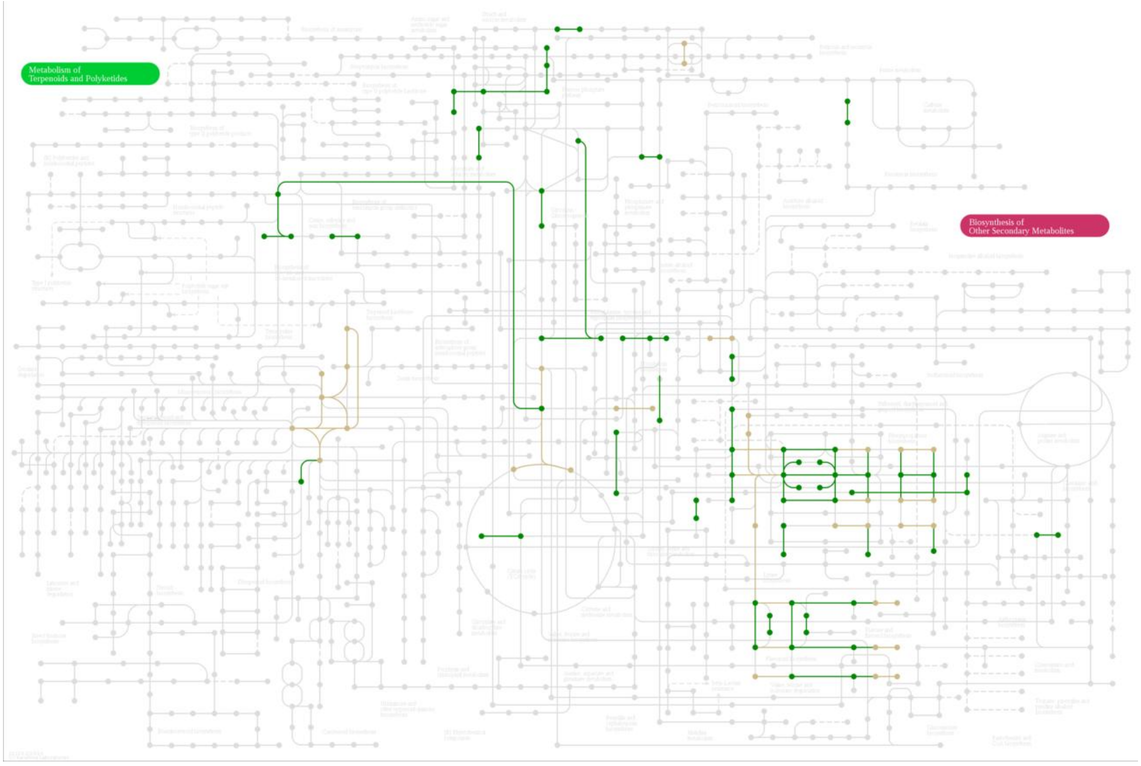

Figure S9. KEGG pathway for the biosynthesis of secondary metabolites (ko01110) including the brown and green-yellow modules.

Methods S1. Metabolite network analysis (WGCNA) script.

```
#Metabolite and Trait data load
getwd();
workingDir = "C:/Users/...";
setwd(workingDir);
library(WGCNA);
options(stringsAsFactors = FALSE);
femData = read.csv("MetaboliteData.csv");
traitData = read.csv("Traits.csv");

femaleSamples1 = rownames(datExpr0);
traitRows1 = match(femaleSamples1, traitData$Sample);
datTraits1 = traitData[traitRows1, -1];
rownames(datTraits1) = traitData[traitRows1, 1];
collectGarbage();
ExprTraits = data.frame(datTraits1$Whorl, datTraits1$Month,
datExpr0)

#Unnecessary data removing and data transposing

datExpr0 = as.data.frame(t(femData[, -c(1)]));
names(datExpr0) = femData$Metabolite;
rownames(datExpr0) = names(femData)[-c(1)];

# Choosing the soft-thresholding power: analysis of network
topology

powers = c(c(1:10), seq(from = 12, to=20, by=2))
sft = pickSoftThreshold(datExpr0, powerVector = powers,
verbose = 5)
write.csv(sft, file="SoftTrehold_Powers_for_Network.csv")
plot(sft$fitIndices[,1], -
sign(sft$fitIndices[,3])*sft$fitIndices[,2],
xlab="Soft Threshold (power)",ylab="Scale Free Topology
Model Fit,signed R^2",type="n",
main = paste("Scale independence"));
text(sft$fitIndices[,1], -
sign(sft$fitIndices[,3])*sft$fitIndices[,2],
labels=powers,cex=cex1,col="red");
abline(h=0.85,col="red")
plot(sft$fitIndices[,1], sft$fitIndices[,5],
xlab="Soft Threshold (power)",ylab="Mean Connectivity",
type="n",
main = paste("Mean connectivity"))
text(sft$fitIndices[,1], sft$fitIndices[,5], labels=powers,
cex=cex1,col="red")
k=softConnectivity(datE=datExpr0,power=6)
scaleFreePlot(k, main="Check scale free topology\n",
pch=19, col="black")
```

```
# Network constrcution

net = blockwiseModules(datExpr0, maxBlockSize = 10000,
power = 6, minModuleSize = 2,
reassignThreshold = 0, mergeCutHeight = 0.25,
numericLabels = TRUE, pamRespectsDendro = FALSE,
saveTOMs = TRUE,
saveTOMFileBase = "TOM1",
verbose = 3, checkMissingData = F)
table(net$colors)
moduleLabels = net$colors
moduleColors = labels2colors(net$colors)
MEs = net$MEs;
geneTree = net$dendrograms[[1]];

#Correlations between gene modules and traits

nGenes = ncol(datExpr0);
nSamples = nrow(datExpr0);
MEs0 = moduleEigengenes(datExpr0, moduleColors)$eigengenes
MEs = orderMEs(MEs0)
moduleTraitCor = cor(MEs, datTraits, use = "p");
moduleTraitPvalue = corPvalueStudent(moduleTraitCor,
nSamples);
textMatrix = paste(signif(moduleTraitCor, 2), "\n(",
signif(moduleTraitPvalue, 1), ")", sep = "");
dim(textMatrix) = dim(moduleTraitCor)
par(mar = c(5, 10, 3, 5));
#par(mar = c(0.7, 1, 0.35, 0.35))
labeledHeatmap(Matrix = moduleTraitCor,
xLabels = names(datTraits),
yLabels = names(MEs),
ySymbols = names(MEs),
colorLabels = FALSE,
colors = blueWhiteRed(50),
textMatrix = textMatrix,
setStdMargins = FALSE,
cex.text = 1.5,
cex.main = 2,
cex.lab = 1.5,
zlim = c(-1,1),
main = paste("Module-trait relationships"))

# Plot the network

dissTOM = 1-TOMsimilarityFromExpr(datExpr0, power = 6);
plotTOM = dissTOM^7;
diag(plotTOM) = NA;
TOMplot(plotTOM, geneTree, moduleColors, main = "Network
heatmap plot", mar=c(9.5, 9.5))
```

```
# Metabolite screening method based on a detailed definition module membership (it can be made for any trait changing to the appropiate "y" value)

NS1=networkScreening(y=datTraits$Whorl, datME=datME, datExpr=datExpr0, oddPower=3, blockSize=2000, minimumSampleSize=4,addMEy=TRUE, removeDiag=FALSE, weightESy=0.5, getQValues=TRUE)

# Output of the results of network screening analysis

GeneResultsNetworkScreening1=data.frame(GeneName=row.names(NS1), NS1)
probes = names(datExpr0)
geneInfo1 = data.frame(name = probes,
                       moduleColor = moduleColors,
                       GeneResultsNetworkScreening1)
write.table(geneInfo1,
file="GeneResultsNetworkScreeningWhorl.csv",
row.names=F,sep=",")
datMEy = data.frame(datTraits$Whorl, datME)
eigengeneSignificance1 = cor(datMEy, datTraits$Whorl);
eigengeneSignificance1[1,1] =
(1+max(eigengeneSignificance1[-1, 1]))/2
eigengeneSignificance1.pvalue =
corPvalueStudent(eigengeneSignificance1, nSamples =
length(datTraits$Whorl))
namesME=names(datMEy)
out1=data.frame(t(data.frame(eigengeneSignificance1,
                             eigengeneSignificance1.pvalue,
namesME, t(datMEy))))
dimnames(out1)[[1]][1]="EigengeneSignificance"
dimnames(out1)[[1]][2]="EigengeneSignificancePvalue"
dimnames(out1)[[1]][3]="ModuleEigengeneName"
dimnames(out1)[[1]][-c(1:3)]=dimnames(datExpr0)[[1]]
write.table(out1,
file="MEResultsNetworkScreeningWhorl.csv", row.names=TRUE,
col.names = TRUE, sep=",")

# Metabolite screening method based on a detailed definition module membership (it can be made for any trait changing to the appropiate "y" value)

NS2=networkScreening(y=datTraits$Month, datME=datME, datExpr=datExpr0, oddPower=3, blockSize=2000, minimumSampleSize=4,addMEy=TRUE, removeDiag=FALSE, weightESy=0.5, getQValues=TRUE)

# Output of the results of network screening analysis
```

```
GeneResultsNetworkScreening2=data.frame(GeneName=row.names(
NS2), NS2)
geneInfo2 = data.frame(name = probes,
                       moduleColor = moduleColors,
                       GeneResultsNetworkScreening2)
write.table(geneInfo2,
file="GeneResultsNetworkScreeningMonth.csv",
row.names=F,sep=",")
datMEy = data.frame(datTraits$Month, datME)
eigengeneSignificance2 = cor(datMEy, datTraits$Month);
eigengeneSignificance2[1,1]                            =
(1+max(eigengeneSignificance2[-1, 1]))/2
eigengeneSignificance2.pvalue                          =
corPvalueStudent(eigengeneSignificance2,    nSamples    =
length(datTraits$Month))
namesME=names(datMEy)
out2=data.frame(t(data.frame(eigengeneSignificance2,
                             eigengeneSignificance2.pvalue,
namesME, t(datMEy))))
dimnames(out2)[[1]][1]="EigengeneSignificance"
dimnames(out2)[[1]][2]="EigengeneSignificancePvalue"
dimnames(out2)[[1]][3]="ModuleEigengeneName"
dimnames(out2)[[1]][-c(1:3)]=dimnames(datExpr0)[[1]]
write.table(out2,
file="MEResultsNetworkScreeningMonth.csv",  row.names=TRUE,
col.names = TRUE, sep=",")
```

Methods S2. Metabolite PCA R script.

```
#Metabolite data load

getwd();
workingDir = "C:/Users/...";
setwd(workingDir);
Data = read.csv("MetaboliteData.csv")
dim(Data)
names(Data)
datExpr1 = as.data.frame(Data[, -c(1)])
dim(datExpr1)
names(datExpr1)
rownames(datExpr1) = Data$Metabolite
rownames(datExpr1)

#PCA calculation

Metabolites_pca <- prcomp(datExpr1)
summary(Metabolites_pca)


#Component plotting

plot(Metabolites_pca, ylim=c(0,200))

#PCA plotting, only metabolites

biplot(Metabolites_pca,var.axes=FALSE,      col=c("black",
"red"), cex=c(1,1),expand=0, xlim=c(-0.35,1))

#PCA plotting, metabolites and samples

biplot(Metabolites_pca,var.axes=FALSE,      col=c("black",
"red"), cex=c(0.5,0.8),expand=1)
```

Methods S3. BABAR script for microarray data normalization.

```
library(babar)
library(limma)
basedir<-"/microarray"
setwd(basedir)
REFERENCEDETECTION="OFF"
NUMBEROFSD<-3
SPAN<-0.3
LOESS<-TRUE
FINALCENTRE<-TRUE
BOXPLOTS=TRUE
bluefiles<-dir(basedir, pattern=".xls$", full.names=TRUE)
bluefiles<-NULL
genepixfiles<-dir(basedir, pattern=".gpr$",
full.names=TRUE)
ratiodata<-babar(bluefiles,genepixfiles)
a<-ratiodata$exprs*(-1)
results<-cbind(ratiodata[["genes"]], a)
write.table(results, file = "c:/microarray/Results.txt",
sep="\t", col.names=NA)
colnames(ratiodata[["exprs"]])
```

Methods S4. Gene network analysis (WGCNA) script.

```
#Microarray and Trait data load

getwd()
workingDir = "C:/Users/..."
setwd(workingDir)
library(WGCNA)
options(stringsAsFactors = FALSE)
femData = read.csv("MicroarrayData1.csv")
traitData = read.csv("Traits1.csv")

femaleSamples1 = rownames(datExpr0);
traitRows1 = match(femaleSamples1, traitData$Sample)
datTraits1 = traitData[traitRows1, -1]
rownames(datTraits1) = traitData[traitRows1, 1]
collectGarbage()
ExprTraits = data.frame(datTraits1$Whorl, datTraits1$Month,
datExpr0)

#Unnecessary data removing and data transposing

datExpr0 = as.data.frame(t(femData[, -c(1)]))
names(datExpr0) = femData$gene;
rownames(datExpr0) = names(femData)[-c(1)]

#Mean expression calcule

meanExpressionByArray=apply( datExpr0,1,mean, na.rm=T)
NumberMissingByArray=apply( is.na(data.frame(datExpr0)),1,
sum)
NumberMissingByArray
barplot(meanExpressionByArray, ylab = "Mean expression",
main ="Mean expression across samples", names.arg =
rownames(datExpr0),
col=rainbow(length(unique(ExprTraits$datTraits1.Whorl)))[as
.factor(ExprTraits$datTraits1.Whorl)], cex.names =
0.6,las=2)

#Verification and removal of genes with too many missing
values

gsg = goodSamplesGenes(datExpr0, verbose = 3)
gsg$allOK
if (!gsg$allOK)
{
if (sum(!gsg$goodGenes)>0)
printFlush(paste("Removing genes:",
paste(names(datExpr0)[!gsg$goodGenes], collapse = ", ")))
if (sum(!gsg$goodSamples)>0)
```

```
printFlush(paste("Removing samples:",
paste(rownames(datExpr0)[!gsg$goodSamples], collapse = ",
")))
datExpr0 = datExpr0[gsg$goodSamples, gsg$goodGenes]
}


#Hierarchical clusterin of the samples

sampleTree = flashClust(dist(datExpr0), method = "average")
par(cex = 0.6);
par(mar = c(1,5,2,0.5))
plot(sampleTree, main = "Sample clustering to detect
outliers", sub="", xlab="", cex.lab = 1.5, cex.axis = 1.5,
cex.main = 2)
abline(h = 25, col = "red")

#Mean expression calcule after gene reomval

meanExpressionByArray=apply( datExpr0,1,mean, na.rm=T)
NumberMissingByArray=apply( is.na(data.frame(datExpr0)),1,
sum)
NumberMissingByArray
par(mar=c(8,4,4,0))
barplot(meanExpressionByArray, ylab = "Mean expression",
main ="Mean expression across samples", names.arg =
rownames(datExpr0),col=rainbow(length(unique(ExprTraits$dat
Traits1.Whorl)))[as.factor(ExprTraits$datTraits1.Whorl)],
cex.names = 0.6,las=2)

# Removal of outlier samples

rownames(datExpr0)
datExpr<-datExpr0[-(47), ]
sampleTree = flashClust(dist(datExpr), method = "average")
par(cex = 0.6);
par(mar = c(1,5,2,0.5))
plot(sampleTree, main = "Sample clustering to detect
outliers", sub="", xlab="", cex.lab = 1.5, cex.axis = 1.5,
cex.main = 2)
abline(h = 25, col = "red")

#Mean expression after gene and sample removals

meanExpressionByArray=apply( datExpr,1,mean, na.rm=T)
NumberMissingByArray=apply( is.na(data.frame(datExpr)),1,
sum)
NumberMissingByArray
femaleSamples = rownames(datExpr)
traitRows = match(femaleSamples, traitData$Sample)
datTraits = traitData[traitRows, -1]
```

```
rownames(datTraits) = traitData[traitRows, 1]
collectGarbage()
ExprTraits1 = data.frame(datTraits$Whorl, datTraits$Month,
datExpr)
par(mar=c(8,4,4,0))
barplot(meanExpressionByArray, ylab = "Mean expression",
main ="Mean expression across samples", names.arg =
rownames(datExpr),col=rainbow(length(unique(ExprTraits1$dat
Traits.Whorl)))[as.factor(ExprTraits1$datTraits.Whorl)],
cex.names = 0.6,las=2)

# Choosing the soft-thresholding power: analysis of network
topology

powers = c(c(1:10), seq(from = 12, to=20, by=2))
sft = pickSoftThreshold(datExpr, powerVector = powers,
verbose = 5)
write.csv(sft, file="SoftTrehold_Powers_for_Network.csv")
sizeGrWindow(9, 5)
par(mfrow = c(1,2))
cex1 = 0.9
plot(sft$fitIndices[,1], -
sign(sft$fitIndices[,3])*sft$fitIndices[,2], xlab="Soft
Threshold (power)",ylab="Scale Free Topology Model
Fit,signed R^2",type="n", main = paste("Scale
independence"))
text(sft$fitIndices[,1], -
sign(sft$fitIndices[,3])*sft$fitIndices[,2],labels=powers,c
ex=cex1,col="red")
abline(h=0.85,col="red")
plot(sft$fitIndices[,1], sft$fitIndices[,5], xlab="Soft
Threshold (power)",ylab="Mean Connectivity", type="n", main
= paste("Mean connectivity"))
text(sft$fitIndices[,1], sft$fitIndices[,5], labels=powers,
cex=cex1,col="red")
k=softConnectivity(datE=datExpr,power=14)
sizeGrWindow(10,5)
par(mfrow=c(1,2))
hist(k, col="darkgrey")
scaleFreePlot(k, main="Check scale free topology\n",
pch=19, col="black")

# Network constrcution

net = blockwiseModules(datExpr, maxBlockSize = 10000, power
= 14, minModuleSize = 30, reassignThreshold = 0,
mergeCutHeight = 0.25, numericLabels = TRUE,
pamRespectsDendro = FALSE, saveTOMs = TRUE, saveTOMFileBase
= "TOM", verbose = 3)
table(net$colors)
sizeGrWindow(12, 9)
```

```
mergedColors = labels2colors(net$colors)
plotDendroAndColors(net$dendrograms[[1]],
mergedColors[net$blockGenes[[1]]], "Module colors",
dendroLabels = FALSE, hang = 0.03, addGuide = TRUE,
guideHang = 0.05)
moduleLabels = net$colors
moduleColors = labels2colors(net$colors)
MEs = net$MEs
geneTree = net$dendrograms[[1]]

#Correlations between gene modules and traits

nGenes = ncol(datExpr)
nSamples = nrow(datExpr)
MEs0 = moduleEigengenes(datExpr, moduleColors)$eigengenes
MEs = orderMEs(MEs0)
moduleTraitCor = cor(MEs, datTraits, use = "p")
moduleTraitPvalue = corPvalueStudent(moduleTraitCor,
nSamples)
sizeGrWindow(10,6)
textMatrix = paste(signif(moduleTraitCor, 2), "\n(",
signif(moduleTraitPvalue, 1), ")", sep = "")
dim(textMatrix) = dim(moduleTraitCor)
par(mar = c(9, 11, 3, 0))
labeledHeatmap(Matrix = moduleTraitCor,xLabels =
names(datTraits),yLabels = names(MEs),ySymbols =
names(MEs),colorLabels = FALSE, colors = blueWhiteRed(50),
textMatrix = textMatrix, setStdMargins = FALSE, cex.text =
0.5, cex.main = 2, cex.lab = 1.2, zlim = c(-1,1),main =
paste("Module-trait relationships"))

#Representing modules by eigengenes and relating eigengenes
to one another

signif(cor(MEs0, use="p"), 2)

#We define a dissimilarity measure between the module
eigengenes that keeps track of the sign of the correlation
#between the module eigengenes, and use it to cluster the
eigengene:
dissimME=(1-t(cor(MEs0, method="p")))/2
hclustdatME=hclust(as.dist(dissimME), method="average" )
par(mfrow=c(1,1))
plot(hclustdatME, main="Clustering tree based of the module
eigengenes")
MEs = orderMEs(MEs0)
moduleTraitCor = cor(MEs, datTraits, use = "p")
moduleTraitPvalue = corPvalueStudent(moduleTraitCor,
nSamples)
```

```
#Pairwise scatter plots of the samples (arrays) along the
module eigengenes (it can be made for any trait changing
"y" value)
plotMEpairs(MEs0,y=datTraits$Whorl, main= "Relationship
between module eigengenes - Whorl", cex.main=2)

# Recalculate module eigengenes

MEas = moduleEigengenes(datExpr, moduleColors, excludeGrey
= FALSE, grey = ifelse(is.numeric(colors), 0,
"orange"))$eigengenes

# Plotting relationships of selected triats with eigengene
modules (it can made for any trait selecting the appropiate
trait in "datTraits$...")
Sucrose = as.data.frame(datTraits$Sucrose)
names(Sucrose) = "Sucrose"
Month = as.data.frame(datTraits$Month)
names(Month) = "Month"
Whorl = as.data.frame(datTraits$Whorl)
names(Whorl) = "Whorl"
Arginine = as.data.frame(datTraits$L.Arginine)
names(Arginine) = "Arginine"
MET = orderMEs(cbind(MEas, Sucrose, Month, Whorl,
Arginine))
sizeGrWindow(5,7.5)
par(cex = 0.9)
plotEigengeneNetworks(MET, heatmapColors=blueWhiteRed(50),
excludeGrey = FALSE, greyLabel = "orange", "", marDendro =
c(0,4,1,2), marHeatmap = c(4,4,1,2), cex.lab = 0.8,
xLabelsAngle= 90)

# Plot the network

dissTOM = 1-TOMsimilarityFromExpr(datExpr, power = 14)
plotTOM = dissTOM^7;
diag(plotTOM) = NA;
sizeGrWindow(9,9)
TOMplot(plotTOM, geneTree, moduleColors, main = "Network
heatmap plot, all genes")

# Plot 400 selceted genes of the network. The image file of
the plot with all genes can be too big.

nGenes = ncol(datExpr)
nSamples = nrow(datExpr)
nSelect = 400
set.seed(10)
select = sample(nGenes, size = nSelect)
selectTOM = dissTOM[select, select]
```

```
selectTree = flashClust(as.dist(selectTOM), method =
"average")
selectColors = moduleColors[select]
sizeGrWindow(9,9)
plotDiss = selectTOM^7
diag(plotDiss) = NA
TOMplot(plotDiss, selectTree, selectColors, main = "Network
heatmap plot, selected genes")

#Multi-dimensional scaling plot of the network

cmd1=cmdscale(as.dist(dissTOM),2)
sizeGrWindow(7, 6)
par(mfrow=c(1,1))
plot(cmd1, col=as.character(moduleColors), main="MDS plot",
xlab="Scaling Dimension 1", ylab="Scaling Dimension 2")

# Recalculate module eigengenes

datME=moduleEigengenes(datExpr,moduleColors)$eigengenes

# Measure of module significance as average gene
significance (it can be made for al the traits changing the
"datTraits~$..." value)

GS1=as.numeric(cor(datTraits$Whorl,datExpr, use="p"))
GeneSignificance1=abs(GS0)
ModuleSignificanceWhorl=tapply(GeneSignificance0,
moduleColors, mean, na.rm=T)
sizeGrWindow(8,7)
par(mar=c(8.3,5,2,2))
plotModuleSignificance(GeneSignificance1,moduleColors,
cex=1.5, cex.main=2, cex.lab=1.5, cex.axis=1.5,
cex.sub=1.5,axis.lty=1, las=3, ylim=c(0,0.7))

# Write the module significance data frame into a file

moduleInfo0 = data.frame( ModuleSignificanceTree,
ModuleSignificanceWhorl, ModuleSignificanceMonth,
ModuleSignificanceAcetate, ModuleSignificanceArginine,
ModuleSignificanceAscorbate, ModuleSignificanceAspartate,
ModuleSignificanceAsparagine, ModuleSignificanceBetaine,
ModuleSignificanceHydroxybutyric,
ModuleSignificanceCholine, ModuleSignificanceCitrate,
ModuleSignificanceCitrulline, ModuleSignificanceCreatine,
ModuleSignificanceFructose, ModuleSignificanceFucose,
ModuleSignificanceGlucuronate,
ModuleSignificanceGlucose.1.phosphate,
ModuleSignificanceGlucose.6.phosphate,
ModuleSignificanceGlucose, ModuleSignificanceGlycine,
```

```
ModuleSignificanceGlyceraldehyde,
ModuleSignificanceCholine,
ModuleSignificanceGlycerophosphocholine,
ModuleSignificanceGlutathione,
ModuleSignificanceIsoleucine,
ModuleSignificanceInositol, ModuleSignificanceLactose,
ModuleSignificanceLeucine, ModuleSignificanceLysine,
ModuleSignificanceMaltose, ModuleSignificanceMannose,
ModuleSignificanceMethionine, ModuleSignificancePinitol,
ModuleSignificanceProline, ModuleSignificanceShikimate,
ModuleSignificanceSuccinate,
ModuleSignificanceSucrose, ModuleSignificanceThreonine,
ModuleSignificanceHydroxyproline, ModuleSignificanceXylose,
ModuleSignificanceUnknown_Sugar, ModuleSignificanceGPC_Cho,
ModuleSignificanceIns_Gly)
names(moduleInfo0) = names(datTraits)
write.table(moduleInfo0,
file="ModuleSignificanceTraits.csv", row.names=T,sep=",")

# Calculate the intramodular connectivity

ADJ1=abs(cor(datExpr,use="p"))^6
Alldegrees1=intramodularConnectivity(ADJ1, moduleColors)
head(Alldegrees1)

#Relationship between gene significance and intramodular
connectivity for Whorl (it can be made for any trait
changing to the appropiate "GeneSignificance..." objet)

colorlevels=unique(moduleColors)
sizeGrWindow(9,6)
par(mfrow=c(4,3))
par(mar = c(4.5,5,4,3))
par(cex=0.5)
for (i in c(1:length(colorlevels)))
{
  whichmodule=colorlevels[[i]]
  restrict1 = (moduleColors==whichmodule)
  verboseScatterplot(Alldegrees1$kWithin[restrict1],
GeneSignificance1[restrict1], col=moduleColors[restrict1],
main=whichmodule, xlab = "Connectivity", ylab = "Gene
Significance", abline = TRUE)
}

#Generalizing intramodular connectivity for all genes on
the array

datKME=signedKME(datExpr, datME, outputColumnName="MM.")

#Relationship between the module membership measures (e.g.
MM.turquoise) and intramodular connectivity (it can be made
```

```
for any trait and module changing to the appropiate
"which.color=..." value)

sizeGrWindow(8,6)
which.color="black";
restrictGenes=moduleColors==which.color
verboseScatterplot(Alldegrees1$kWithin[
restrictGenes],(datKME[restrictGenes, paste("MM.",
which.color, sep="")])^6, col=which.color,
xlab="Intramodular Connectivity",ylab="(Module
Membership)^6", main=which.color)

# Gene screening method based on a detailed definition
module membership (it can be made for any trait changing to
the appropiate "y" value)

NS1=networkScreening(y=datTraits$Whorl, datME=datME,
datExpr=datExpr, oddPower=3, blockSize=2000,
minimumSampleSize=4,addMEy=TRUE, removeDiag=FALSE,
weightESy=0.5, getQValues=TRUE)

# Comparing the weighted correlation with the standard
Pearson correlation
# Form a data frame containing standard and network
screening results and plot the comparison

CorPrediction1=data.frame(GS1,NS1$cor.Weighted)
cor.Weighted1=NS1$cor.Weighted
sizeGrWindow(8, 6)
par(mar = c(4,5,3,3))
par(cex=1.5)
verboseScatterplot(cor.Weighted1, GS1,main="Network-based
weighted correlation versus Pearson
correlation\n",col=moduleColors, cex.main = 1.2)
abline(0,1)

# Output of the results of network screening analysis

GeneResultsNetworkScreening1=data.frame(GeneName=row.names(
NS1), NS1)
annot = read.csv(file = "AnnotationPinarray2.csv")
dim(annot)
names(annot)
probes = names(datExpr)
probes2annot = match(probes, annot$Name)
sum(is.na(probes2annot))
geneInfo1 = data.frame(name = probes, Description =
annot$Description[probes2annot], SustainPine3 =
annot$SustainPine3[probes2annot],ID =
annot$ID[probes2annot],GO_Terms =
```

```
annot$GO_terms_Sma3[probes2annot],moduleColor =
moduleColors,GeneResultsNetworkScreening1)
write.table(geneInfo1,
file="GeneResultsNetworkScreeningWhorl.csv",
row.names=F,sep=",")

# Save the output of eigengene information

datMEy = data.frame(datTraits$Whorl, datME)
eigengeneSignificance1 = cor(datMEy, datTraits$Whorl)
eigengeneSignificance1[1,1] =
(1+max(eigengeneSignificance1[-1, 1]))/2
eigengeneSignificance1.pvalue =
corPvalueStudent(eigengeneSignificance1, nSamples =
length(datTraits$Whorl))
namesME=names(datMEy)
out1=data.frame(t(data.frame(eigengeneSignificance1,
eigengeneSignificance1.pvalue, namesME, t(datMEy))))
dimnames(out1)[[1]][1]="EigengeneSignificance"
dimnames(out1)[[1]][2]="EigengeneSignificancePvalue"
dimnames(out1)[[1]][3]="ModuleEigengeneName"
dimnames(out1)[[1]][-c(1:3)]=dimnames(datExpr)[[1]]
write.table(out1,
file="MEResultsNetworkScreeningWhorl.csv", row.names=TRUE,
col.names = TRUE, sep=",")

# Plotthe signed and unsigned correlations of the 30 top
genes for a trait

topList=rank(NS1$p.Weighted,ties.method="first")<=30
gene.names= names(datExpr)[topList]
sizeGrWindow(7,7)
par(cex.main=0.5)
plotNetworkHeatmap(datExpr, plotGenes = gene.names,
networkType="signed", useTOM=FALSE, power=14, main="A.
signed correlations")
sizeGrWindow(7,7)
par(cex.main=0.5)
plotNetworkHeatmap(datExpr, plotGenes = gene.names,
networkType="unsigned", useTOM=FALSE, power=14, main="B.
unsigned correlations")
sizeGrWindow(7,7)
par(cex.main=0.5)
plotNetworkHeatmap(datExpr, plotGenes =
gene.names,networkType="signed", useTOM=TRUE, power=14,
main="C. TOM in a signed network")
sizeGrWindow(7,7)
par(cex.main=0.5)
plotNetworkHeatmap(datExpr, plotGenes =
gene.names,networkType="unsigned", useTOM=TRUE, power=14,
main="D. TOM in an unsigned network")
```

```
# Network output file for VisAnt (it can be made for any
module cahnging the "module=..." value)

module = "black";
probes = names(datExpr)
inModule = (moduleColors==module)
modProbes = probes[inModule]
modTOM = TOM[inModule, inModule]
dimnames(modTOM) = list(modProbes, modProbes)
vis = exportNetworkToVisANT(modTOM, file =
paste("VisANTInput-", module, ".txt", sep=""), weighted =
TRUE, threshold = 0, probeToGene = data.frame(annot$Name,
annot$SustainPine3))

# Network output file for VisAnt only for 100 genes (it can
be made for any module cahnging the "module=..." value)

module = "black"
probes = names(datExpr)
inModule = (moduleColors==module)
modProbes = probes[inModule]
modTOM = TOM[inModule, inModule]
dimnames(modTOM) = list(modProbes, modProbes)
nTop = 100
IMConn = softConnectivity(datExpr[, modProbes])
top = (rank(-IMConn) <= nTop)
vis = exportNetworkToVisANT(modTOM[top, top], file =
paste("VisANTInput-", module, "-top100.txt", sep=""),
weighted = TRUE, threshold = 0, probeToGene =
data.frame(annot$Name, annot$SustainPine3))

# Network output file for Cytoscape (it can be made for any
module cahnging the "module=..." value)

module = "black"
probes = names(datExpr)
inModule = is.finite(match(moduleColors, module))
modProbes = probes[inModule]
modGenes = annot$Name[match(modProbes, annot$SustainPine3)]
modTOM = TOM[inModule, inModule]
dimnames(modTOM) = list(modProbes, modProbes)
cyt = exportNetworkToCytoscape(modTOM, edgeFile =
paste("CytoscapeInput-edges-", paste(module, collapse="-"),
".txt", sep=""), nodeFile = paste("CytoscapeInput-nodes-",
paste(module, collapse="-"), ".txt", sep=""), weighted =
TRUE, threshold = 0, nodeNames = modProbes, altNodeNames =
modGenes, nodeAttr = moduleColors[inModule])
```

```
# Network output file for Cytoscape only for 100 genes (it
can be made for any module cahnging the "module=..." value)

module = "black"
probes = names(datExpr)
inModule = is.finite(match(moduleColors, module))
modProbes = probes[inModule]
modGenes = annot$Name[match(modProbes, annot$SustainPine3)]
modTOM = TOM[inModule, inModule]
dimnames(modTOM) = list(modProbes, modProbes)
nTop = 100;
IMConn = softConnectivity(datExpr[, modProbes])
top = (rank(-IMConn) <= nTop)
cyt = exportNetworkToCytoscape(modTOM[top, top], edgeFile =
paste("CytoscapeInput-edges-", paste(module, collapse="-"),
"-top100.txt", sep=""), nodeFile = paste("CytoscapeInput-
nodes-", paste(module, collapse="-"), "-top100.txt",
sep=""), weighted = TRUE, threshold = 0, nodeNames =
modProbes, altNodeNames = modGenes, nodeAttr =
moduleColors[inModule])
```
